# An Interpretable, Data‐Driven, Hierarchical Multi‐Domain Fusion Framework for Classification and Motor Function Scoring in Chronic Ankle Instability

**DOI:** 10.1002/advs.77212

**Published:** 2026-08-31

**Authors:** Tianle Jie, Datao Xu, Zhifeng Zhou, Bas Van Hooren, Huiyu Zhou, Yi Yuan, Xiangli Gao, Monèm Jemni, Liangliang Xiang, Meizi Wang, Justin Fernandez, Tibor J. Goda, Yaodong Gu

**Affiliations:** ^1^ Faculty of Sports Science Ningbo University Ningbo China; ^2^ Doctoral School on Safety and Security Sciences Óbuda University Budapest Hungary; ^3^ Department of Nutrition and Movement Sciences NUTRIM School of Nutrition and Translational Research in Metabolism Maastricht University Medical Centre+ Maastricht The Netherlands; ^4^ Research Academy of Medicine Combining Sports Ningbo No. 2 Hospital Ningbo China; ^5^ Department of Kinesiology Hungarian University of Sports Sciences Budapest Hungary; ^6^ Centre for Mental Health Research University of Cambridge Cambridge Cambridgeshire UK; ^7^ The Carrick Institute of Neuroscience Cape Coral Florida USA; ^8^ KTH MoveAbility Lab Department of Engineering Mechanics KTH Royal Institute of Technology Stockholm Sweden; ^9^ Auckland Bioengineering Institute University of Auckland Auckland New Zealand; ^10^ Department of Biomedical Engineering Faculty of Engineering Hong Kong Polytechnic University Hong Kong China

**Keywords:** chronic ankle instability, hierarchical multi‐domain fusion, model interpretability, personalized motor function assessment, sports medicine

## Abstract

Chronic ankle instability (CAI) is a common sports‐related musculoskeletal disorder characterized by recurrent sprains and neuromuscular control deficits. It affects a wide range of individuals, from recreational to elite athletes, and poses a substantial healthcare burden. This study proposes an AI‐enabled digital twin framework for sports health applications, offering both interpretability and clinical deployability. The framework identifies CAI and enables subtype stratification using a wearable electromyography (EMG) sensor‐driven hierarchical multi‐domain fusion model, generates fine‐grained motor function scores through a probabilistic modeling approach, and further translates the generated scores into clinically interpretable functional stratification to support rehabilitation assessment. SHapley Additive exPlanations (SHAP) ‐ based interpretability reveals key predictive biomarkers underlying model decisions, establishing a transparent and closed‐loop framework for personalized rehabilitation. Validation on 150 participants, including CAI patients and healthy controls, confirms robust classification performance (Accuracy = 98.50%, AUC = 0.99), reliable discrimination of CAI subtypes (Accuracy = 87.70%, macro F1‐score = 87.20%), and strong concordance between the generated scores and the clinical gold‐standard scale (*r* = −0.908,  p  <  0.001). This non‐invasive, personalized assessment framework supports long‐term rehabilitation management of chronic conditions, offering an innovative and cost‐effective digital health solution for sports medicine.

## Graphical abstract

An AI‐enabled digital twin framework integrates wearable EMG sensing with hierarchical multi‐domain fusion to classify chronic ankle instability, distinguish clinically relevant subtypes, and generate continuous motor function scores. Clinically interpretable functional stratification and SHAP‐based biomarker analysis provide transparent decision support, advancing personalized rehabilitation assessment and digital health management.

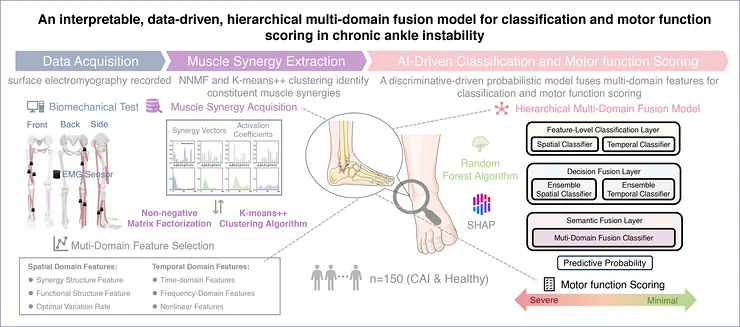

## 1 Introduction

Ankle sprains are among the most common injuries in sports medicine, with an incidence of 16%–40% in physically active populations [1] and comprising 15%–20% of all lower‐extremity sports traumas [2]. Approximately 40% of individuals sustaining a first‐episode sprain will progress to chronic ankle instability (CAI) [3]. This instability has become a significant public‐health concern within the global burden of musculoskeletal diseases [4]. Health‐economic analyses indicate that the acute direct medical expenses associated with ankle sprains, together with secondary productivity losses, impose a substantial financial burden [5]. For example, data from nationwide modeling in the Netherlands estimate annual costs of approximately €187 million, of which productivity loss accounts for nearly 80%. At the individual level, patients typically require around 2.5 weeks of sick leave, and up to 10% remain unable to resume work by 6 weeks [6]. Further, CAI markedly increases the risk of post‐traumatic osteoarthritis (PTOA) [7]. Longitudinal clinical follow‐ups have shown that a notable minority of individuals with longstanding instability develop radiographic degenerative changes over subsequent decades, with reported rates in the order of approximately 10%–15% after long‐term observation [8, 9]. Consequently, early, standardized, and individualized rehabilitation interventions are the preferred first‐line strategy to maximize restoration of joint mechanical stability and neuromuscular control, thereby preventing the onset of chronic instability and degenerative changes.

In current clinical practice, ankle sprain rehabilitation has evolved into a comprehensive treatment framework that integrates multimodal interventions, including neuromuscular control training [10] and proprioceptive rehabilitation [11], alongside emerging assistive technologies like exoskeleton robotics [12, 13]. While these interventions have demonstrated significant efficacy in promoting functional recovery, there remain notable limitations in the objective quantification of functional progress throughout the rehabilitation process. Commonly used clinical assessment tools, such as the Foot and Ankle Ability Measure (FAAM) [14] and the American Orthopaedic Foot and Ankle Society (AOFAS) Ankle–Hindfoot Score [15], rely primarily on ordinal scales and are heavily dependent on clinicians’ subjective judgment, leading to variability in outcomes. Moreover, these instruments are prone to ceiling effects during the early stages of recovery, where patients with higher functional capacity quickly reach the upper limit of the scale, rendering subtle but clinically meaningful changes difficult to detect [16]. Emerging evidence further indicates that even when patients report satisfactory subjective outcomes, advanced motion capture systems can still identify aberrant movement patterns within the lower limb kinetic chain, underscoring the limitations of traditional subjective tools in capturing the complexity of motor function restoration [10, 17, 18]. Therefore, there is an urgent need to develop assessment approaches that are highly sensitive, objective, and capable of comprehensively quantifying the recovery of ankle biomechanics and neuromuscular function. This shift would promote a paradigm transition from subjective clinical judgment to objective, data‐driven analysis, ultimately enabling multidimensional visualization of neuromuscular–biomechanical–functional recovery trajectories.

Previous studies have primarily relied on joint kinematics [19], kinetics [20], or clinical measures such as muscle strength and range of motion [21] to classify CAI using conventional machine learning models. These approaches, however, exhibit fundamental limitations. Kinematic and kinetic parameters capture only the external manifestation of movement performance. Individuals with CAI frequently present with adaptive biomechanical alterations [22], and prior work has shown that, to compensate for impaired proprioception and reduced ankle stability, they often adopt hip‐ or knee‐dominant strategies during landing tasks [23, 24, 25]. Such compensatory behaviors may mask deficits specific to the ankle and diminish the informativeness of these parameters, thereby restricting the achievable classification accuracy. Traditional clinical and biomechanical indicators also tend to emphasize isolated joint function or single‐domain features [19], which are insufficient to characterize the multifactorial and heterogeneous presentation of CAI. As a result, machine learning models trained solely on these metrics often capture superficial statistical patterns rather than underlying physiological mechanisms, leading to limited generalizability and unstable performance when confronted with new participants [26]. Although some studies have explored CAI identification using medical imaging and reported high classification accuracy [27, 28], imaging‐based methods face distinct constraints. Structural imaging is well suited for detecting morphological abnormalities but is poorly aligned with the longitudinal monitoring of functional recovery. In addition, such models provide little insight into neuromuscular control strategies, limiting their relevance for functional assessment or rehabilitation planning. Discrepancies between imaging findings and patients' perceived instability or functional impairment further reduce the clinical value of imaging‐driven models [29].

Integrating muscle synergy analysis within a multi‐domain hierarchical classification framework provides a compelling alternative. Hierarchical fusion of complementary domains has demonstrated superior performance in functional state quantification, high‐level feature representation, and model generalization compared with traditional feature‐based pipelines [30, 31, 32]. Muscle synergy analysis offers direct insight into the coordination strategies implemented by the central nervous system and yields physiologically interpretable features that extend beyond what can be captured by kinematic or kinetic metrics alone [33, 34, 35]. By decomposing surface electromyography (sEMG) into spatial components (synergy vectors) and temporal components (activation coefficients), synergy‐based models enable multidimensional characterization of neuromuscular control [36]. Time‐domain descriptors such as mean activation amplitude or onset/offset timing can be informative, yet frequency‐domain analysis may reveal deficits that temporal metrics fail to detect. This is particularly relevant for CAI subgroups that preserve typical spatial synergy structure but exhibit perturbed activation timing, or vice versa [37]. These observations highlight the value of multi‐domain feature fusion for modeling neuromuscular dysfunction in CAI.

In the context of rehabilitation monitoring, current research has largely followed two methodological directions. One approach employs inertial sensing systems to extract gait‐based kinematic and kinetic features and then interprets the output probabilities of machine learning classifiers as surrogate indicators of recovery status [13]. The evaluative logic of this approach, however, remains constrained by the dichotomous distinction between healthy and pathological movement. The probability value generated by such models represents only a softened version of a discrete classification outcome rather than a quantitative metric positioned along a continuum of functional capability. More importantly, these models lack physiological interpretability. Their scalar outputs cannot be meaningfully linked to neuromuscular control deficits such as delayed activation responses or impaired multi‐joint coordination, which are hallmark features of CAI. The resulting opacity restricts clinical applicability and limits the utility of these models in guiding individualized rehabilitation. A second line of research aims to predict rehabilitation success using patient‐reported outcome measures (PROMs). These approaches face inherent limitations in criterion validity because the operational definition of recovery depends entirely on subjective perceptions [38]. This dependency introduces susceptibility to recall bias, social desirability effects, and placebo influences, creating possible dissociations between reported improvement and objective functional change. Furthermore, the variables typically included in such models—demographic profiles, medical history, and static clinical signs—lack quantitative evaluation of movement‐control quality. Consequently, these models describe relationships between subjective indicators rather than establishing a reproducible evaluation framework grounded in objective neuromuscular function and measurable functional recovery. Taken together, these limitations underscore the need for a physiologically interpretable, objectively grounded, and functionally continuous approach to rehabilitation assessment, motivating the development of synergy‐informed, multi‐domain evaluation models.

A digital‐twin framework offers considerable promise for addressing these challenges [39]. By mapping muscle‐synergy features extracted from subject‐specific sEMG into a computational predictive model, the system enables synchronous updates of the participant's physical movement patterns and their virtual representation within a shared state space. Interpretive analyses of the digital twin, including feature‐importance profiling, make it possible to identify the synergy modules that contribute most to functional impairment and to quantify deviations in specific synergy vectors. These deviations can be translated into actionable clinical feedback, providing clinicians and rehabilitation specialists with real‐time, quantitative, and individualized diagnostic insights and enabling the development of targeted intervention programs. This enhances both the precision and the timeliness of rehabilitation strategies. With further refinement of the underlying machine learning models, the system could operate reliably outside traditional clinical settings, transforming CAI monitoring from episodic, clinic‐based assessments to continuous, ecologically valid evaluation. Such an approach may reduce the need for repeated clinical visits and imaging examinations, promote greater consistency in patients’ daily behaviors, and improve engagement in rehabilitation activities.

Therefore, this study aims to bridge a critical gap between accurate identification of CAI and personalized rehabilitation management by developing and validating a novel digital twin framework. The overall methodological pipeline of this proposed framework is systematically depicted in Figure 1. Specifically, the proposed system leverages real‐time data acquired from wearable sEMG sensors to drive a hierarchical multi‐domain fusion model for high‐precision CAI classification, while further distinguishing clinically relevant CAI subtypes. Furthermore, a probabilistic modeling approach enables the generation of fine‐grained, individualized motor function scores, which can be further translated into clinically interpretable functional severity levels to facilitate rehabilitation assessment. Through SHapley Additive exPlanations (SHAP)‐based explainability analysis, key neuromuscular predictive biomarkers influencing model decisions are identified, establishing a transparent, closed‐loop digital health decision support system that links precise classification, dynamic functional assessment, functional severity stratification, and mechanistic interpretation. This digital twin model dynamically tracks patient‐specific motor recovery trajectories, empowering rehabilitation specialists to adjust intervention strategies in real time, and ultimately offering a more efficient and precise rehabilitation pathway for both general and elite athletic populations.

**FIGURE 1:**
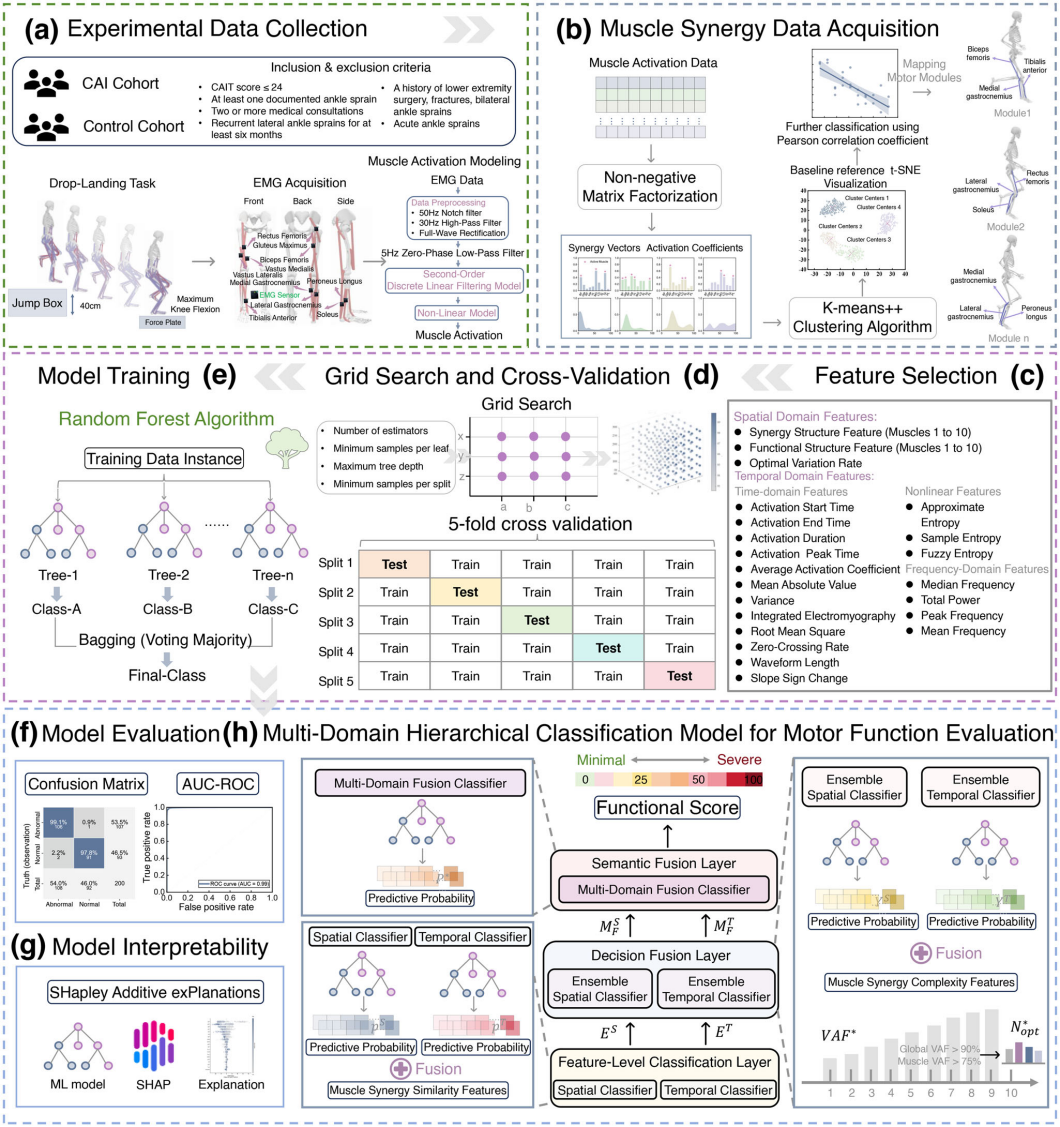
Schematic of the proposed workflow pipeline. (a) Participant screening and sEMG acquisition and preprocessing; (b) Extraction of muscle synergy patterns via NNMF and K‐means++ clustering; (c) Muti‐domain feature extraction from muscle synergy profiles; (d) Hyperparameter optimization using grid search with cross‐validation; (e) Training of random forest (RF) classifiers on selected features; (f) Evaluation of model performance using classification metrics; (g) Model interpretability analysis using SHAP; (h) Generation of motor function scores based on probabilistic modeling with a hierarchical multi‐domain fusion classifier.

## 2 Results

### 2.1 Muscle Synergy Structure and Interpretation

We applied the non‐negative matrix factorization (NNMF) algorithm to the preprocessed sEMG signals to extract synergy vectors and their corresponding activation coefficients. According to the predefined criterion, the optimal number of muscle synergies was determined as the smallest synergy order at which the global variance accounted for (VAF) exceeded 90% and all local muscle VAFs surpassed 75%. As illustrated in Figure 2a–d, and detailed in Table S1, the CAI, functional ankle instability (FAI), mechanical ankle instability (MAI), and healthy control groups all met these thresholds primarily when the number of synergies reached four. Further statistical analysis of the individual optimal synergy numbers is presented in Figure 2e. The FAI group exhibited the highest number of synergies, which was significantly higher than both the healthy controls (*p* < 0.001) and the MAI group (*p* < 0.05). The overall CAI group also showed a significantly higher optimal number of muscle synergies compared to healthy controls (*p* < 0.001). All synergy vectors derived from the healthy group were subjected to cluster analysis to identify representative neuromuscular control modules. To determine the optimal number of clusters, we calculated the within‐cluster sum of squares (WCSS) and the average silhouette coefficient under different cluster conditions. As illustrated in Figure 2f, the WCSS exhibited a noticeable inflection point at four clusters, beyond which the rate of decrease substantially slowed. Similarly, Figure 2g shows that the silhouette coefficient reached its peak when the cluster number was four, indicating that the clustering structure was most appropriate under this condition. Accordingly, we selected four clusters as the optimal solution, suggesting the presence of four principal neuromuscular control patterns during the landing task.

**FIGURE 2:**
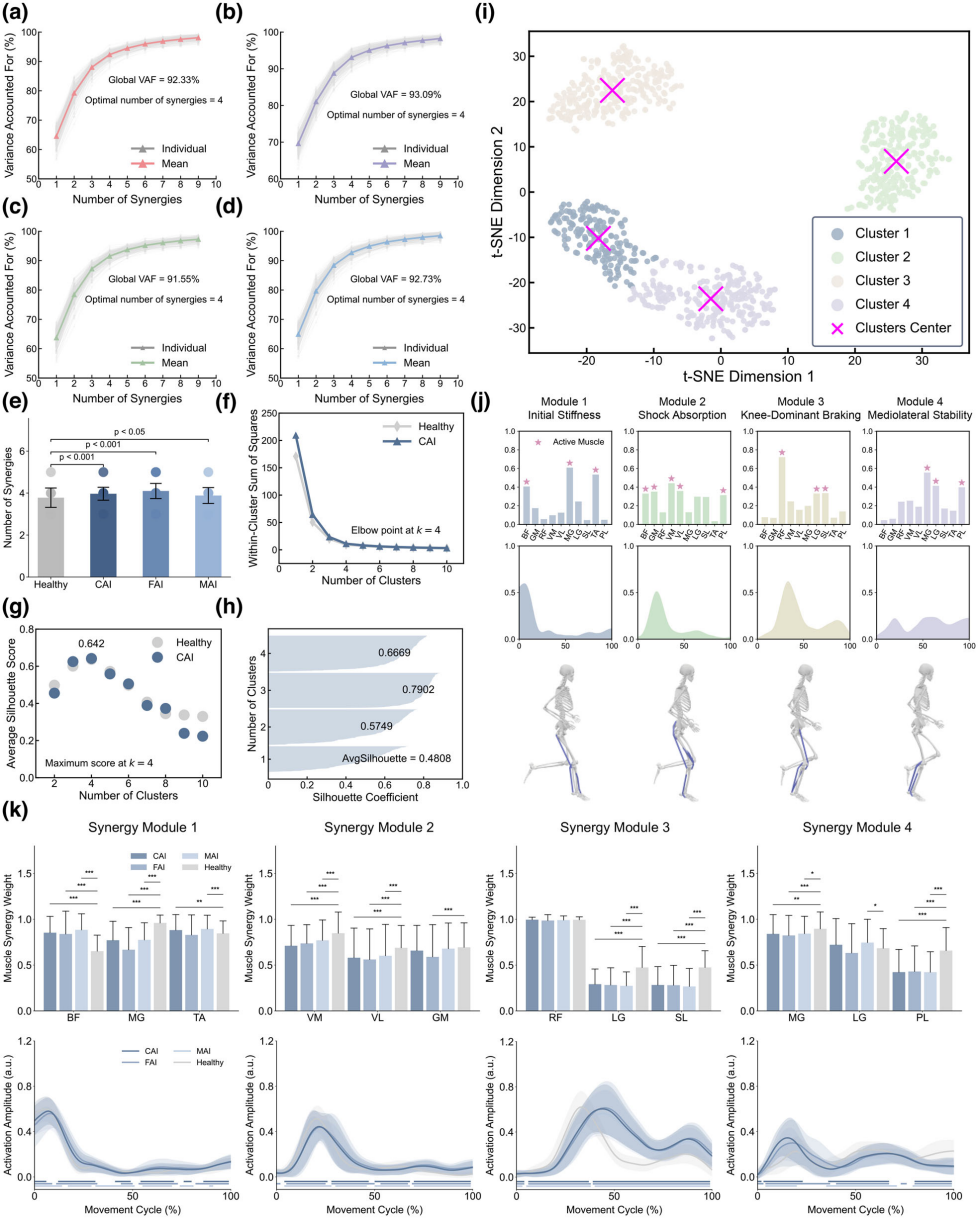
Visualization of NNMF‐based muscle synergy extraction and clustering results. (a) VAF across synergy numbers in the CAI group. (b) Global VAF across synergy numbers in the healthy group. (c) Global VAF across synergy numbers in the FAI group. (d) Global VAF across synergy numbers in the MAI group. (e) Comparison of average synergy numbers among Healthy, CAI, FAI, and MAI groups. (f) Elbow curve of WCSS. (g) Average silhouette coefficient across cluster numbers. (h) Silhouette coefficients of all samples under the optimal clustering solution. (i) Two‐dimensional embedding of synergy vectors using t‐SNE. (j) Visualization of the four clustered motor modules. (k) Group‐averaged synergy weights and activation amplitudes among the CAI, MAI, FAI, and healthy groups.

Figure 2h presents the silhouette coefficients for all samples, indicating well‐separated clusters with minimal evidence of misclassification, thus confirming the effectiveness and reliability of the clustering results. To further evaluate and visualize the clustering performance, we employed t‐distributed Stochastic Neighbor Embedding (t‐SNE) to reduce the dimensionality of the high‐dimensional synergy vectors. The detailed parameter settings of the t‐SNE algorithm are provided in Table S2. As shown in Figure 2i, all data points were clearly separated into four non‐overlapping clusters in the two‐dimensional space. Each cluster was compact and had distinct boundaries, and the between‐cluster separability was high, collectively demonstrating the robustness and discriminative capability of the clustering model.

To elucidate their functional relevance during the drop‐landing task, these four clustered motor modules were classified based on their principal muscle weights and biomechanical roles (Figure 2j). Module 1 (Initial Stiffness), dominated by the biceps femoris (BF), medial gastrocnemius (MG), and tibialis anterior (TA), is primarily responsible for pre‐landing preparation and rapid joint stiffening during initial ground contact. Module 2 (Shock Absorption), characterized by the activation of proximal muscles (BF, gluteus maximus (GM), vastus medialis (VM), vastus lateralis (VL), and peroneus longus (PL)), represents a coordinated eccentric control strategy to attenuate impact forces through knee and hip flexion. Module 3 (Knee‐Dominant Braking), driven by the rectus femoris (RF), lateral gastrocnemius (LG), and soleus (SL), facilitates deceleration and anterior‐posterior balance regulation. Finally, Module 4 (Mediolateral Stability), involving the MG, LG, and PL, is a distal module crucial for ankle inversion‐eversion control and mediolateral postural stabilization. Further comparisons of the extracted motor modules revealed that the CAI, FAI, and MAI cohorts employed distinctly altered neuromuscular control strategies compared to healthy individuals (Figure 2k). Specifically, the instability groups exhibited significant deviations in both spatial muscle weightings and continuous temporal activation dynamics throughout the drop‐landing. A comprehensive statistical breakdown of these spatial synergy structures and temporal activation profiles is detailed in Text S1 and Figure S1.

### 2.2 Analysis of Extracted Muscle Synergy Features

In this study, three spatial‐domain features and nineteen temporal‐domain features were constructed based on muscle synergy data extracted using NNMF (Figure S2). Across these features, the majority exhibited discernible differences between the CAI and healthy groups (Table S3), providing a feature space suitable for subsequent classification model development. Building upon this, a set of higher‐order features reflecting the complexity of muscle synergies was further extracted, including the optimal number of synergies, synergy structure similarity, and VAF (Table S4). Through multi‐domain feature fusion, the model was able to construct more distinct decision boundaries, potentially enhancing its sensitivity and accuracy in identifying pathological motor control patterns.

### 2.3 Performance of Classification Models

To gain a deeper understanding of how model performance is influenced by hyperparameter settings, we first evaluated the relative importance of key hyperparameters using results from a grid search (Figure S3a). The analysis revealed that the minimum samples per leaf consistently exhibited high importance across all models. In addition, the maximum tree depth was particularly influential in the spatial and temporal classifiers, while the number of estimators had a more pronounced impact on ensemble‐based models. Based on this evaluation, the three most critical hyperparameters—maximum tree depth, minimum samples per leaf, and number of estimators—were selected for further analysis. A three‐dimensional hyperparameter performance landscape was constructed to visualize how combinations of these parameters affect classification accuracy (Figure S3b). The visualization results showed that the spatial classifier achieved higher accuracy when the maximum tree depth was relatively large (8–10), the minimum samples per leaf was small (1–10), and the number of estimators was high (200–300). The ensemble spatial classifier exhibited more stable performance within similar parameter ranges, with optimal regions concentrated around a maximum tree depth of 6–10, a minimum samples per leaf of less than 10, and a number of estimators greater than 200, demonstrating enhanced robustness. In contrast, the temporal classifier showed more scattered and unstable accuracy distributions, suggesting that temporal features alone may be insufficient for reliable classification. When ensemble strategies were applied, the ensemble temporal classifier showed substantial performance improvement under similar hyperparameter settings. The multi‐domain fusion classifier achieved the best overall performance among all models. It not only yielded the highest accuracy levels but also exhibited a densely concentrated high‐performance region, indicating strong hyperparameter stability. The optimal configuration was found with a maximum tree depth between 6 and 10, a minimum samples per leaf of less than 5, and a number of estimators greater than 200. The optimal hyperparameter configurations for each classification model, as determined through grid search with cross‐validation, are summarized in Table 1.

**TABLE 1:** Specific hyperparameter configurations of all classification models.

Hyperparameters	Spatial Classifier	Ensemble Spatial Classifier	Temporal Classifier	Ensemble Temporal Classifier	Multi‐Domain Fusion Classifier
Number of estimators	200	50	150	300	50
Maximum tree depth	8	6	12	6	6
Minimum samples per leaf	5	5	5	5	5
Minimum samples per split	20	5	5	5	5

Figure 3a,b present the confusion matrices and receiver operating characteristic (ROC) curves of the five evaluated classification models, with corresponding performance metrics detailed in Table 2. Among the base models, the spatial classifier demonstrated superior discriminative performance compared with the temporal classifier, achieving higher accuracy (88.3%, compared with 85.1%), F1‐score (89.3%, compared with 85.1%), and area under the curve (AUC) (0.95, compared with 0.93). Ensemble strategies further improved predictive performance. Notably, the ensemble spatial classifier improved upon its base counterpart by 4.0% in accuracy and 2.7% in F1‐score, achieving an overall accuracy of 92.3%. It also resulted in fewer false‐negative (13 cases) and false‐positive predictions (14 cases) compared with the ensemble temporal model (22 and 17 cases, respectively). Ultimately, the multi‐domain fusion classifier demonstrated the best overall performance. By integrating both spatial and temporal domains, it achieved an accuracy of 98.5% and an F1‐score of 98.4%, representing a 6.4% improvement in F1‐score over the best‐performing ensemble model. The AUC reached 0.99, and the confusion matrix showed only 1 false negative and 2 false positives, representing the lowest misclassification rate among all evaluated models.

**FIGURE 3:**
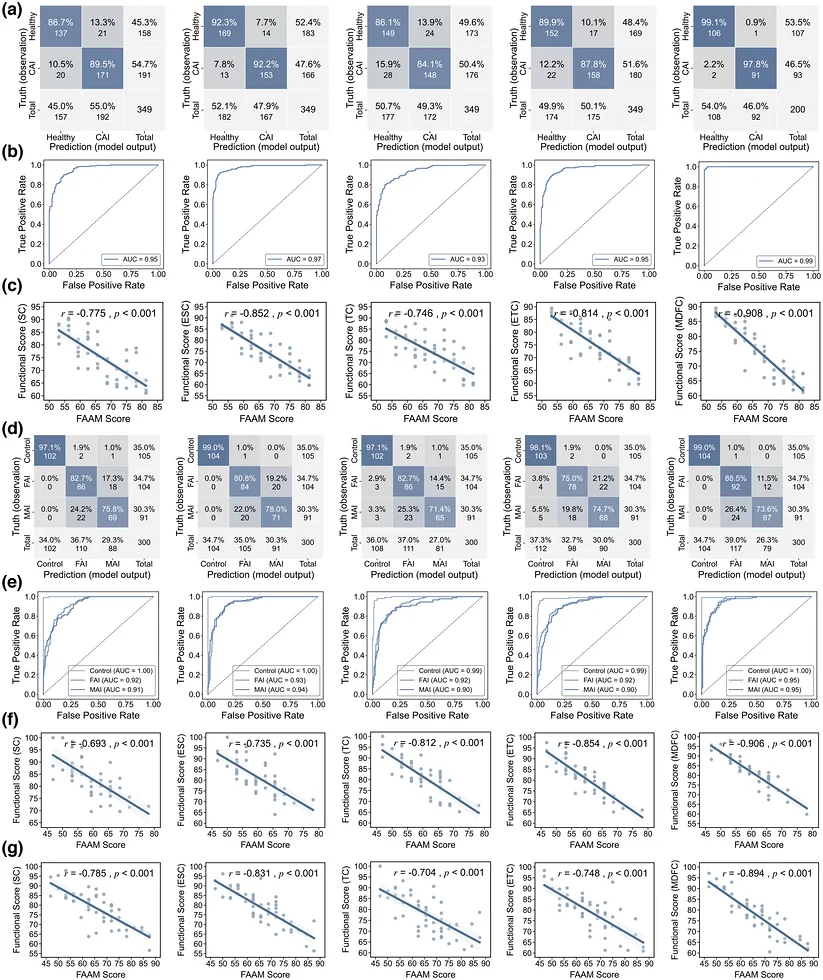
Visualization of classification performance and functional score validation across five models. (a) Confusion matrices for the binary classification task distinguishing healthy controls from individuals with CAI. (b) Receiver operating characteristic (ROC) curves for the binary classification task. (c) Correlation analyses between model‐derived functional scores and clinical FAAM scores for the overall CAI cohort. (d) Confusion matrices for the multi‐class classification task stratifying healthy controls, FAI, and MAI. (e) ROC curves for the multi‐class classification task. (f) Correlation analyses between model‐derived functional scores and FAAM scores for the specific pathological subgroup of FAI. (g) Correlation analyses between model‐derived functional scores and FAAM scores for the specific pathological subgroup of MAI. Note: Models are presented from left to right in the following order: Spatial Classifier, Ensemble Spatial Classifier, Temporal Classifier, Ensemble Temporal Classifier, and Multi‐domain Fusion Classifier.

**TABLE 2:** Classification accuracy, precision, recall, and F1 score (%) of all classifiers.

Performance metrics	Spatial Classifier	Ensemble Spatial Classifier	Temporal Classifier	Ensemble Temporal Classifier	Multi‐Domain Fusion Classifier
Binary Classification					
Accuracy	88.3	92.3	85.1	88.8	98.5
Precision	89.1	91.6	86.1	90.3	98.9
Recall	89.5	92.2	84.1	87.8	97.8
F1‐Measure	89.3	92.0	85.1	89.0	98.4
Multi‐class Classification					
Accuracy	85.7	86.3	84.3	83.0	87.7
Macro‐Precision	85.5	86.0	84.1	82.4	87.8
Macro‐Recall	85.2	86.0	83.8	82.6	87.1
Macro F1‐ Measure	85.3	86.0	83.8	82.4	87.2

For the three‐class classification task, Figure 3d,e, alongside Table 2, summarize the quantitative performance. Consistent with the binary classification task, all architectures identified healthy controls with a true positive rate above 97.0%. For discrimination between the two pathological subtypes, the base spatial classifier achieved a higher macro F1‐score (85.3%, compared with 83.8%) and recall for MAI cases (75.8%, compared with 71.4%) than the temporal model. Applying ensemble strategies produced distinct effects: the ensemble spatial classifier improved its macro F1‐score to 86.0% and increased the AUC for MAI detection from 0.91 to 0.94, whereas the ensemble temporal model exhibited a decrease in macro F1‐score to 82.4%. Finally, the multi‐domain fusion classifier achieved the best overall performance in the multi‐class evaluation, reaching an accuracy of 87.7% and a macro F1‐score of 87.2%. For the pathological subtypes, this fusion architecture achieved recall rates of 88.5% for FAI and 73.6% for MAI, with corresponding AUC values of 0.95 for both categories.

### 2.4 Performance Validation of the Functional Scoring Models

To validate the effectiveness of the proposed functional scoring framework, this study performed correlation analyses between the model‐derived functional scores and FAAM scores. Figure 3c illustrates the correlation results for the five classification models. Overall, all models showed significant negative correlations with the FAAM scores (p < 0.001), indicating that lower FAAM scores were associated with higher predicted impairment scores from the models. Among the single‐domain classifiers, the spatial classifier achieved a stronger correlation with the FAAM score (*r* = −0.775) compared to the temporal classifier (*r* = −0.746). The ensemble models further improved the correlation strength to *r* = −0.852 for the ensemble spatial classifier and *r* = −0.814 for the ensemble temporal classifier. The multi‐domain fusion classifier demonstrated the best performance, achieving the highest correlation with FAAM scores (*r* = −0.908), which outperformed both the single‐domain base and ensemble models.

Building upon the overall correlation results, we further stratified the analysis to evaluate the framework's scoring fidelity within the specific pathological subgroups of FAI and MAI (Figures 3f and 5g). All evaluated models demonstrated significant negative correlations with the FAAM scores in both pathological subtypes (p < 0.001). Among the base models, the temporal classifier exhibited a stronger correlation with the FAAM score for the FAI subgroup compared to the spatial classifier (*r* = −0.812 vs. *r* = −0.693). Conversely, for the MAI subgroup, the spatial classifier yielded a higher correlation than the temporal model (*r* = −0.785 vs. *r* = −0.704). The application of ensemble strategies consistently improved the correlation strength across both cohorts. For the FAI group, the correlation coefficients increased to −0.735 for the ensemble spatial classifier and −0.854 for the ensemble temporal classifier. Similarly, for the MAI group, the ensemble models achieved higher correlations of −0.831 (spatial) and −0.854 (temporal). Ultimately, the multi‐domain fusion classifier achieved the highest correlation with the FAAM scores in both clinical subtypes. The multi‐domain fusion classifier reached *r* = −0.906 for the FAI group and *r* = −0.894 for the MAI group, outperforming all single‐domain base and ensemble models.

### 2.5 Interpretability Analysis of the Hierarchical Classification and Functional Scoring Models

In the spatial classifier (Figure 4a), the top five contributing features ranked by SHAP values were synergy structure features (*F_W_*) of the lateral gastrocnemius ($F_{w}^{\mathrm{LG}}$), medial gastrocnemius ($F_{w}^{\mathrm{MG}}$), optimal variation rate (*OVR*), biceps femoris ($F_{w}^{\mathrm{BF}}$), and vastus lateralis ($F_{w}^{\mathrm{VL}}$). Notably, most of these features showed a tendency whereby lower feature values were more indicative of pathological conditions. The temporal classifier primarily captured the dynamic temporal characteristics of muscle synergies (Figure 4b). Its top five SHAP‐ranked features were peak frequency (PF), variance (VAR), time‐to‐peak (*T_peak_*), start time (*T_start_*), and median frequency (MDF). Among them, PF exhibited the strongest positive contribution to pathological classification, while *T_peak_* showed a negative impact, suggesting that earlier occurrence of peak activation may be associated with compensatory motor strategies. In the ensemble classification stage (Figure 4c,d), the classifier output probability consistently ranked first in SHAP importance, demonstrating the highest contribution to model predictions. The SHAP values showed a wide distribution with a clear positive trend in the high‐probability range, indicating that the decision confidence of the classifier played a dominant role in driving the final outputs. Synergy similarity features also exhibited substantial discriminative power, with SHAP values increasing alongside feature magnitude. In the final multi‐domain fusion model (Figure 4e), the predicted probabilities derived from the ensemble spatial and temporal classifiers occupied the top two SHAP ranks, underscoring their primary role in cross‐domain decision integration. In parallel, global muscle synergy complexity features, particularly the optimal number of synergies and the VAF by major synergy components, demonstrated notable contributions.

**FIGURE 4:**
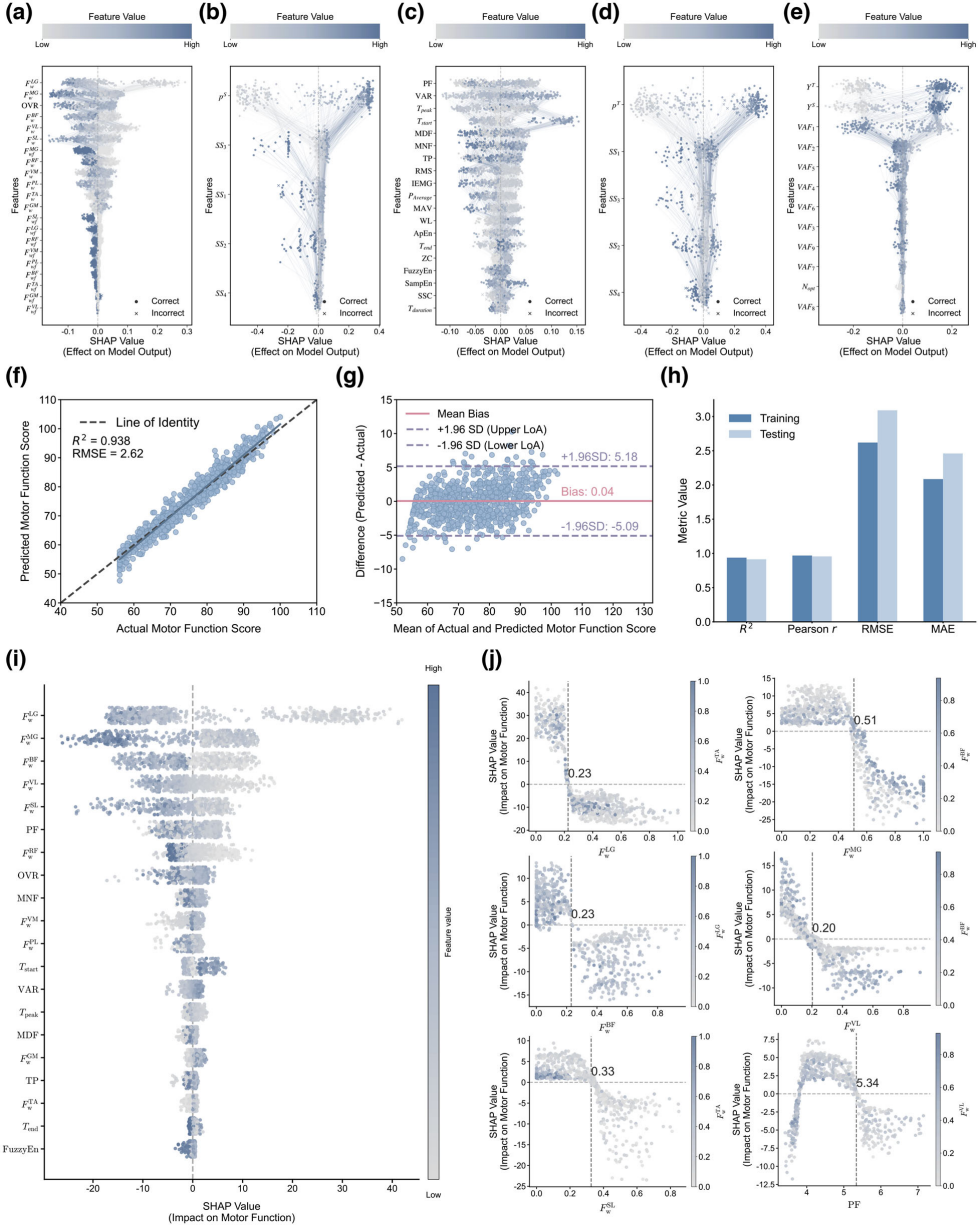
Performance and SHAP‐based interpretability analysis of the hierarchical multi‐domain classification and continuous motor function scoring framework. (a) SHAP summary plot of the base classifier in the spatial domain. (b) SHAP summary plot of the ensemble spatial classifier. (c) SHAP summary plot of the base classifier in the temporal domain. (d) SHAP summary plot of the ensemble temporal classifier. (e) SHAP summary plot of the hierarchical multi‐domain fusion classifier. (f) Scatter plot comparing actual versus predicted motor function scores. (g) Bland‐Altman plot of the predicted motor function scores. (h) Comparison of predictive performance metrics between training and testing datasets. (i) Global SHAP summary plot of neuromuscular features for continuous motor function scoring. (j) SHAP dependence plots of key neuromuscular indicators. Note: *p^S^* and *p^T^* represent the predicted probabilities from the base spatial and temporal classifiers, respectively; *Y^S^* and *Y^T^* denote the decision‐level predicted probabilities from the ensemble spatial and temporal classifiers; *SS*_1_ −*SS*_4_ represent the similarity scores corresponding to the four reference motor modules; *VAF*_1_ −*VAF*_9_ represent the variance accounted for across synergy orders 1 to 9 during the decomposition process; *N_opt_* represents the optimal number of synergies; *F_w_*: synergy structure feature; *F_wf_*: functional structure feature; OVR: optimal variation rate; PF: peak frequency; BF: biceps femoris; GM: gluteus maximus; RF: rectus femoris; VM: vastus medialis; VL: vastus lateralis; MG: medial gastrocnemius; LG: lateral gastrocnemius; SL: soleus; TA: tibialis anterior; PL: peroneus longus.

To further evaluate the efficacy of the continuous motor function scoring framework, we assessed the predictive performance of the surrogate model (Figure 4f–h). The regression analysis (Figure 4f) demonstrated a highly accurate prediction of motor function scores, yielding a coefficient of determination (R^2^) of 0.938 and a root mean square error (RMSE) of 2.62. Bland‐Altman analysis (Figure 4g) further corroborated this high agreement, revealing minimal mean bias and narrow 95% limits of agreement. Moreover, the model demonstrated robust generalization without evidence of overfitting, as indicated by the highly consistent performance metrics (R^2^, Pearson r, RMSE, and mean absolute error (MAE)) across both the training and testing datasets (Figure 4h).

SHAP summary analysis demonstrated the directional impact of key features on the continuous functional score (Figure 4i). The results identified the reduction in distal muscle synergies as a primary factor driving up the functional score. For major calf and thigh features, such as the $F_{w}^{\mathrm{LG}}$, $F_{w}^{\mathrm{VL}}$, and $F_{w}^{\mathrm{RF}}$, reduced feature values were associated with positive SHAP values. Conversely, the $F_{w}^{\mathrm{GM}}$ and $F_{w}^{\mathrm{TA}}$ exhibited an opposite trend, with higher feature values contributing to higher functional scores. Furthermore, the directional impact of certain features was non‐monotonic. Specifically, although elevated values of the $F_{w}^{\mathrm{MG}}$ were associated with negative SHAP values, the region of positive SHAP values contained an overlapping distribution of both high and low feature values. A similar overlapping distribution was observed for the $F_{w}^{\mathrm{PL}}$ and several time‐ and frequency‐domain features.

SHAP dependence plots were utilized to quantitatively characterize the non‐linear threshold effects and feature interactions influencing the continuous functional score (Figure 4j). A consistent non‐linear pattern emerged among the primary muscle synergy features: as the feature values decreased below specific inflection points, their corresponding SHAP values increased sharply, driving the model output toward higher scores. These critical thresholds were identified at 0.23 for the $F_{w}^{\mathrm{LG}}$, 0.51 for the $F_{w}^{\mathrm{MG}}$, 0.33 for the $F_{w}^{\mathrm{SL}}$, and 0.20 for the $F_{w}^{\mathrm{VL}}$.In contrast, the PF exhibited a distinctly biphasic non‐linear trend. Its contribution to the functional score reached a positive peak in the intermediate range but decreased sharply when the value exceeded 5.34 or dropped significantly below this range. Furthermore, for most features, the interacting features exhibited highly overlapping and heterogeneous distributions across the identified thresholds. However, a distinct interaction pattern was observed in the $F_{w}^{\mathrm{SL}}$ plot. Specifically, when $F_{w}^{\mathrm{SL}}$ exceeded the threshold of 0.33, the corresponding SHAP values became negative, indicating a downward contribution to the functional score. Under this condition, $F_{w}^{\mathrm{TA}}$ values were predominantly low.

### 2.6 Clinical Thresholds, Functional Grading, and Discriminant Validity of the Continuous Motor Function Score

The continuous motor function score demonstrated robust discriminant validity, effectively differentiating the CAI cohort from healthy controls (*p* < 0.001; Figure 5a). Probability density estimation revealed distinct distribution patterns with minimal overlap: scores for healthy subjects clustered in the lower range (20–40), whereas CAI subjects exhibited a bimodal distribution in the higher range (60–90) (Figure 5b). Accordingly, ROC analysis yielded an outstanding AUC of 0.969 (Figure 5c), with an optimal diagnostic cut‐off of 62.0 achieving high sensitivity (0.98) and specificity (0.90). Furthermore, the functional scores exhibited a strong negative linear correlation with clinical FAAM scores (Pearson *r* = −0.909, p < 0.001; Figure 5d). By mapping established clinical benchmarks onto this continuous metric, we delineated specific severity intervals (Figure 5e). The threshold for mild impairment (corresponding to a FAAM score of 90) was identified at 53.7 (95% prediction interval (PI): 45.6–61.7), while the boundary for severe impairment (FAAM score of 75) was determined at 67.6 (95% PI: 60.0–75.2). Utilizing these thresholds to stratify the cohort into healthy, mild, and severe subgroups revealed significant, step‐wise variations in raw muscle synergy features and electromyographic metrics (*p* < 0.001; Figure 5f), thereby validating the physiological relevance of the defined clinical grades.

**FIGURE 5:**
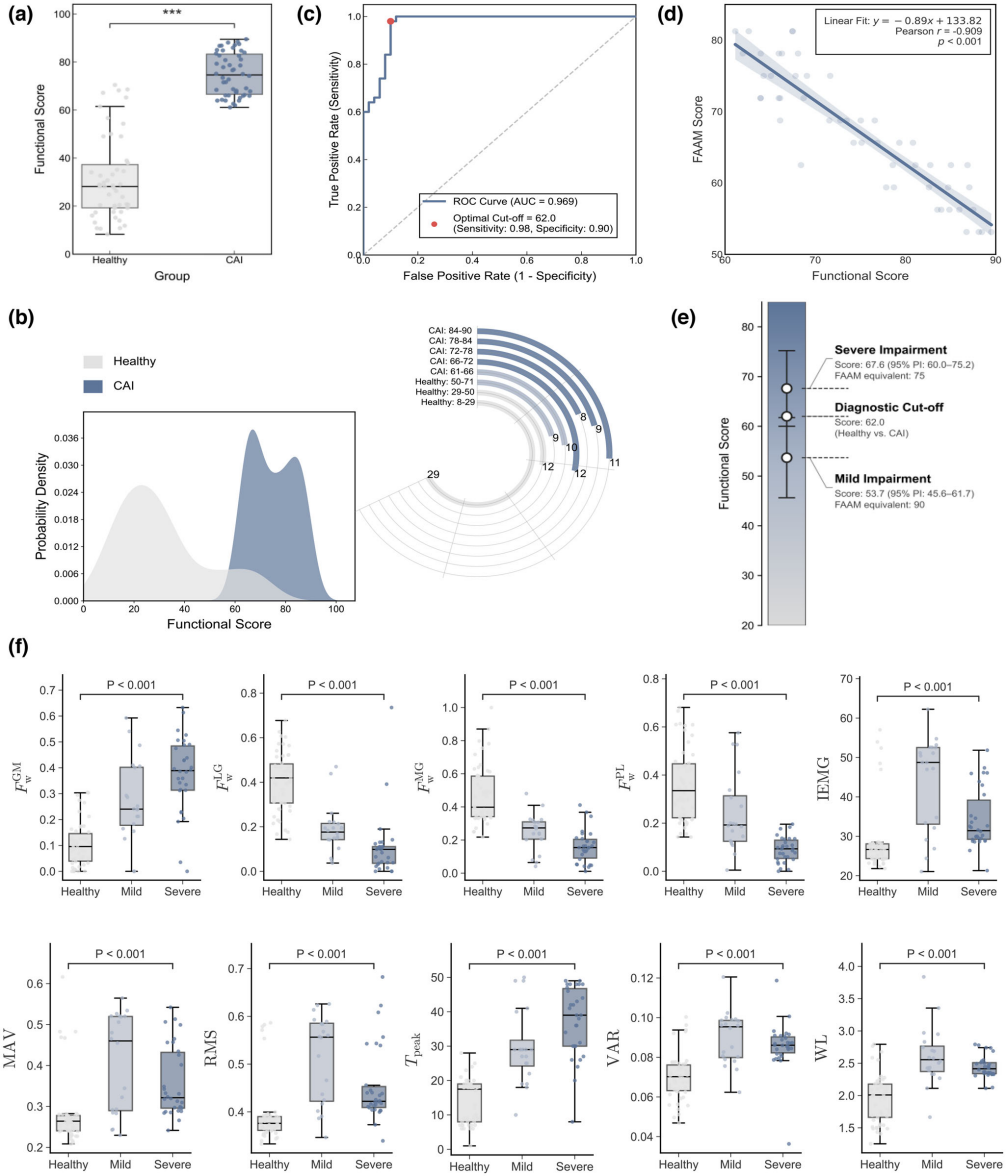
Diagnostic evaluation and functional grading of the continuous motor function scoring framework. (a) Comparison of functional scores between healthy and CAI cohorts. (b) Probability density distributions of the functional scores. (c) ROC curve of the functional score. (d) Linear correlation between functional scores and clinical FAAM scores. (e) Severity stratification thresholds derived from clinical FAAM benchmarks. (f) Comparisons of key features across the stratified subgroups. Note: FAAM: Foot and Ankle Ability Measure; PI: prediction interval; $F_{w}^{GM}$: gluteus maximus synergy structure feature; $F_{w}^{LG}$: lateral gastrocnemius synergy structure feature; $F_{w}^{MG}$: medial gastrocnemius synergy structure feature; $F_{w}^{PL}$: peroneus longus synergy structure feature; IEMG: integrated electromyography; MAV: mean absolute value; RMS: root mean square; *T_peak_*: time to peak activation; VAR: variance; WL: waveform length.

## 3 Discussion

In this study, we developed an integrated intelligent assessment framework that combines accurate classification, interpretable feature analysis, and continuous functional quantification for CAI. By using a hierarchical multi‐domain fusion model driven by wearable sEMG signals, the proposed framework enabled accurate identification of CAI and its clinical subtypes while providing a continuous representation of lower‐limb motor function. Compared with conventional assessments that rely on laboratory‐based biomechanical measurements, imaging techniques, or intermittent clinical evaluations, this approach provides a practical solution for long‐term neuromuscular monitoring and individualized functional assessment outside clinical environments.

A major finding of this study is that spatial and temporal neuromuscular features provide complementary information for characterizing CAI‐related motor control deficits. Our results showed that spatial classifiers exhibited greater stability than temporal classifiers for multi‐class CAI subtyping (macro F1‐score: 85.3% versus 83.8%), as detailed in Figure 2d and Table 2. This advantage may be attributed to the physiological characteristics of muscle synergies. As low‐dimensional and modular representations of motor control generated by the central nervous system, muscle synergies are relatively robust against the variability commonly observed in sEMG signals and subject‐specific movement strategies [40, 41]. In contrast, temporal activation features provide detailed information regarding the timing characteristics of muscle recruitment but are more susceptible to transient fluctuations in movement execution and inter‐individual variability, which may reduce their reliability when used independently [42]. The different responses of spatial and temporal features to ensemble strategies further highlight the complementary nature of these two domains. Probability‐based ensemble learning further enhanced the stability of spatial representations, achieving a multi‐class macro F1 of 86.0% and an MAI detection AUC of 0.94 (Figure 2e, Table 2), which suggests that integrating multiple predictions can reduce variability in synergy‐related features [43, 44]. However, this improvement was absent in the temporal domain, where the macro F1‐score instead degraded slightly to 82.4%. This is potentially because temporal activation patterns contain greater sensitivity to short‐term physiological fluctuations and compensatory movement strategies. These findings support the necessity of integrating multiple neuromuscular domains rather than relying on a single feature representation for CAI assessment.

By combining spatial synergy characteristics with temporal activation dynamics, the multi‐domain fusion classifier achieved high accuracy in both binary (98.5%) and multi‐class (87.7%) evaluations. This robust performance provided additional insights into the heterogeneous mechanisms underlying CAI subtypes. Notably, the fusion model exhibited higher sensitivity for FAI compared to MAI (recall of 88.5% versus 73.6%). This difference is consistent with the distinct clinical characteristics of these two conditions. FAI is primarily associated with impaired proprioceptive function and altered sensorimotor control, which may be reflected by abnormal neuromuscular activation patterns captured by the extracted biomarkers [45]. In contrast, MAI is mainly characterized by structural ligamentous laxity. Although mechanical instability can induce compensatory neuromuscular adaptations during movement, the primary structural impairment may not be fully represented by sEMG‐based features alone [46]. Therefore, the higher sensitivity observed for FAI suggests that the proposed framework may be particularly valuable for identifying neuromuscular control deficits, rather than simply detecting general abnormalities in movement patterns.

When compared with existing CAI diagnostic approaches, our framework provides several methodological advantages (Table S5). Existing approaches primarily follow two technical paths: one relying on conventional biomechanical features such as motion trajectories [19], joint angle variations [20], plantar pressure distributions [13], and muscle strength indicators [21]; the other leveraging medical imaging or multimodal sensor data, including full gait cycle kinematic time‐series and medical images from ultrasound or magnetic resonance imaging (MRI) [13, 28, 47]. While these methods achieve robust binary classification, they uniformly treat CAI as a homogeneous pathology. This monolithic perspective overlooks the divergent rehabilitation pathways required for FAI versus MAI. By jointly modeling spatial synergy structures and temporal dynamics, our approach extends beyond traditional binary screening.

To evaluate the clinical relevance of the generated continuous motor function scores, we examined their association with the widely used FAAM scale (Figure 2c,f,g). Although both base classifiers showed significant negative correlations with FAAM scores in the overall CAI cohort (*p* < 0.001), subgroup analysis revealed distinct sensitivities of different neuromuscular domains. In the FAI subgroup, temporal features showed a stronger association with patient‐reported functional limitations, suggesting that dynamic muscle activation patterns may better reflect impaired sensorimotor control. In contrast, spatial synergy features exhibited a stronger relationship with functional status in the MAI subgroup, indicating that muscle coordination structures may capture compensatory motor strategies associated with mechanical instability. By integrating these complementary domains, the multi‐domain fusion model reduced the limitations of individual feature representations and achieved the strongest agreement with FAAM scores across all subgroups (*r* = −0.906 for FAI; *r* = −0.894 for MAI). These findings demonstrate that the proposed framework can generate a continuous functional measure capable of capturing multiple dimensions of neuromuscular impairment.

We further compared this continuous functional measure with existing approaches used for CAI rehabilitation assessment. Current evaluation strategies primarily rely on discrete classification outcomes, pre‐ and post‐intervention comparisons, or subjective clinical questionnaires [13, 38]. While these methods have demonstrated utility in endpoint outcome assessment and clinical decision‐making, they typically produce discrete labels or binary probabilities and are primarily designed for static, cross‐sectional classification. As such, they have not been extensively validated for tracking individual‐level functional changes over time, a gap that motivates the development of a framework capable of repeated, sensitive, and interpretable assessments. To facilitate the translation of continuous model outputs into clinically interpretable information, we established a data‐driven diagnostic threshold based on the distribution of the generated functional scores (Figure 5b,c). This threshold provides an objective reference for identifying functional impairment levels and may reduce limitations associated with self‐reported assessments, including recall bias and ceiling effects. Building on this threshold‐based interpretation, we further calibrated the continuous model scores against clinically relevant FAAM reference points using linear regression (Figure 5d,e). By aligning functional score ranges with FAAM‐based impairment categories, including reference points corresponding to mild and severe functional limitations, the proposed approach converts complex neuromuscular measurements into an interpretable grading system. Importantly, the validity of these thresholds was further supported by physiological changes observed in the underlying neuromuscular features. When participants were stratified according to the derived severity intervals, muscle synergy characteristics and electromyographic features demonstrated progressive alterations across functional levels (Figure 5f).

To interpret the model predictions and identify clinically meaningful neuromuscular markers, we performed SHAP analysis across the proposed framework. In the spatial classification model, the most influential features were the muscle synergy magnitudes and their directional consistency (Figure 4a). Notably, lower values in distal muscle features, particularly the medial and lateral gastrocnemius ($F_{w}^{LG}$ and $F_{w}^{MG}$), increased the likelihood of CAI classification. This observation aligns with clinical evidence showing reduced activation of the posterior calf in patients with ankle instability [48, 49], suggesting that alterations in distal muscle synergies may represent important markers of impaired dynamic control. Similar patterns were observed in proximal muscle synergies, including the biceps femoris and vastus lateralis ($F_{w}^{BF}$ and $F_{w}^{VL}$). This highlight altered multi‐muscle coordination, supporting the concept that ankle instability affects not only local joint mechanics but also global lower‐limb coordination during dynamic tasks [24, 25, 35, 50]. Furthermore, the contribution of OVR, where reduced directional consistency was associated with CAI classification, suggests decreased organization of synergy patterns. This may reflect reduced consistency in coordinating multiple muscles during functional tasks.

In the temporal model, key features included *T_start_*, *T_peak_*, PF, and MDF were identified as major contributors to model prediction (Figure 4c). Higher *T_start_* and *T_peak_* values showed positive SHAP contributions toward CAI, indicating that delayed muscle activation timing contributes to the identification of individuals with impaired neuromuscular control. Such delayed responses are particularly relevant for muscles involved in rapid postural stabilization, including the tibialis anterior and peroneus longus [51, 52]. In addition, lower frequency‐related features (PF and MDF) contributed positively to CAI classification, suggesting that spectral characteristics of sEMG signals may provide additional information regarding altered neuromuscular function. These changes may be associated with differences in muscle activation strategies and motor unit recruitment patterns reported in individuals with chronic ankle instability [37]. From a rehabilitation perspective, these interpretable temporal and spectral markers may provide objective information for designing targeted interventions, such as emphasizing rapid response training for delayed activation patterns or endurance‐oriented exercises for reduced activation capacity.

SHAP analysis of the continuous functional score further provided insights into the relationship between neuromuscular characteristics and functional impairment severity (Figure 4i). Feature dependence analysis revealed nonlinear associations between key synergy‐related features and the predicted functional score (Figure 4j). Specifically, decreases in the primary synergy weights of the gastrocnemius muscles ($F_{w}^{LG}$, $F_{w}^{MG}$) below specific ranges (approximately 0.23 and 0.51, respectively) were associated with a greater contribution toward functional impairment. These nonlinear patterns suggest that the relationship between neuromuscular alterations and functional limitations is not simply proportional. Instead, progressive reductions in muscle synergy contributions may become increasingly relevant once compensatory control strategies are insufficient to maintain functional stability. Therefore, these feature ranges may serve as potential reference points for monitoring changes in neuromuscular function during rehabilitation. However, longitudinal studies are required to determine whether these thresholds represent clinically meaningful transition points during recovery. The interaction analysis further revealed complex relationships among neuromuscular features. For example, PF exhibited a nonlinear contribution pattern, with its influence on functional impairment being greater within an intermediate range than at extreme values. This pattern may reflect different neuromuscular adaptation strategies associated with chronic instability. In addition, interactions between features, such as the relationship between soleus and tibialis anterior synergy contributions, suggest coordinated adjustments in muscle activation during functional tasks. These interpretable outputs extend the generated functional score from a single numerical index to a more detailed neuromuscular profile. By identifying the specific features and interactions associated with functional impairment, the framework may support individualized rehabilitation planning. For example, when reduced coordination between the soleus and tibialis anterior is identified, rehabilitation strategies may incorporate balance and dynamic control exercises that emphasize coordinated activation of these muscles rather than relying solely on isolated strengthening.

While the framework exhibits strong performance in classification accuracy, physiological interpretability, and individualized scoring, several methodological and translational limitations warrant further investigation in future work. First, although cross‐validation and standardized acquisition protocols were employed to minimize potential bias, the current sample size remains relatively limited. Given the substantial heterogeneity among CAI patients in terms of age distribution, functional status, and compensatory strategies, the generalizability of the proposed model requires further validation on large‐scale, multi‐center datasets encompassing diverse rehabilitation stages. Furthermore, we acknowledge that the current study was cross‐sectional in design and did not directly capture the continuous evolution of functional recovery in CAI patients. However, the proposed framework was designed with longitudinal applicability in mind. Its wearable EMG‐based input enables low‐burden, high‐frequency repeated measurements, while the continuous 0–100 scoring output, rather than discrete classification labels, provides enhanced sensitivity to subtle functional changes. Moreover, the SHAP‐based interpretability framework may facilitate the identification of how specific neuromuscular features evolve throughout rehabilitation. A prospective cohort study with repeated assessments is therefore planned as the next step to empirically validate the framework's sensitivity to clinically meaningful functional improvements over time. Second, although SHAP analysis significantly improved the interpretability of feature contributions, its explanatory outcomes are inherently dependent on the underlying model architecture and the definition of input variables, which may introduce potential biases. Future research could integrate complementary explanation methods and causal inference frameworks to cross‐validate the identified risk factors, thereby enhancing interpretive robustness and clinical credibility. Lastly, while this study preliminarily integrated multi‐domain feature fusion and hierarchical classification mechanisms, the current implementation remains research‐oriented and script‐based, lacking a high degree of modularity and software integration. Future efforts will focus on modularizing and encapsulating the predictive model into a portable software tool tailored for clinical professionals. This will include interactive visualization interfaces, automated report generation, and user‐friendly data processing pipelines to facilitate a seamless transition from algorithmic development to clinical deployment.

## 4 Conclusion

In summary, this study proposes a cutting‐edge intelligent evaluation framework with high interpretability and clinical deployability for sports health applications, aiming at the precise identification of CAI and individualized motor function scoring. The framework employs a wearable sensor data‐driven hierarchical multi‐domain feature fusion classification model to identify CAI while distinguishing clinically relevant CAI subtypes, and introduces a probability‐driven, high‐resolution, and fine‐grained lower‐limb motor function scoring approach. The continuous motor function scores can be further translated into clinically interpretable functional severity levels, providing an objective reference for functional stratification and rehabilitation assessment. Through SHAP‐based interpretability, key neuromuscular predictive biomarkers driving model decisions are revealed, constructing a closed‐loop and transparent personalized medical solution from accurate classification and motor function scoring to physiological mechanism feedback. According to our results, the model demonstrates robust classification accuracy, reliable subtype discrimination, and the generated motor function scores show high consistency with clinical gold‐standard scales on external validation sets. This work has the potential to effectively reduce the medical resource burden caused by repeated diagnosis and treatment following ankle sprains, meet the needs of dynamic tracking of motor function changes in chronic diseases related to neuromuscular control impairments, and provide an intelligent rehabilitation monitoring tool for refined digital health management ranging from elite athletes to individuals undergoing chronic disease rehabilitation.

## 5 Methods

### 5.1 Participants

Participants completed the self‐administered Cumberland Ankle Instability Tool (CAIT). Demographic characteristics, anthropometric measurements, injury history (number of lateral ankle sprains), and clinical examination outcomes, including anterior talar translation and talar tilt differences, were recorded and are summarized in Table 3. A total of 150 participants meeting the inclusion criteria were retrospectively enrolled (Yongjiang Cohort), comprising 100 patients with unilateral CAI and 50 healthy controls. The CAI cohort was further stratified into 50 patients with MAI and 50 patients with FAI based on clinical physical examinations. MAI was diagnosed by the presence of objective mechanical laxity in the lateral ankle ligaments. Specifically, the joint laxity was quantitatively assessed using an instrumented ankle arthrometer administered by a senior sports medicine physician. A positive anterior drawer test was defined as an anterior talar translation of > 3 mm compared to the contralateral limb, and a positive talar tilt test was defined as a difference of > 5° in inversion laxity relative to the uninjured side. Conversely, FAI was characterized by self‐reported recurrent sprains and episodes of joint “giving way” combined with compromised perceived ankle stability, in the strict absence of objective mechanical laxity upon clinical evaluation. Healthy controls had no history of any ankle sprains.

**TABLE 3:** Participant demographics.

	CAI (n = 100) Mean (SD)	FAI (n = 50) Mean (SD)	MAI (n = 50) Mean (SD)	Healthy (n = 50) Mean (SD)
Age(year)	24.0(1.91)	24.3(1.74)	23.7(2.03)	23.3(3.03)
Weight (kg)	78.2(6.07)	79.8(5.85)	77.7(5.25)	76.8(2.14)
Height(cm)	180(5.77)	181(5.74)	180(5.85)	182(4.79)
CAIT score	20.0(3.40)	19.1(3.27)	18.8(3.55)	27.5(1.64)
Number of lateral ankle sprains	3.14(1.84)	3.06(1.93)	3.21(1.75)	/
Anterior talar translation difference (mm)	3.14(1.74)	1.60(0.69)	4.69(0.85)	/
Talar tilt difference (°)	5.01(2.85)	2.41(0.99)	7.60(1.34)	/

Based on the International Ankle Consortium (IAC) consensus statement, the inclusion criteria for CAI patients were:(1) CAIT score ≤ 24. (2) At least one documented ankle sprain, with two or more medical consultations as a result. (3) Recurrent lateral ankle sprains for at least six months, or concerns about ankle dysfunction, sensory instability, and giving way. Exclusion criteria included:(1) A history of lower extremity surgery, fractures, bilateral ankle sprains, or other serious medical conditions. (2) Acute ankle sprains, defined as injuries occurring within the preceding 3 days (72 h), were excluded to avoid enrolling participants with active acute‐phase symptoms [53]. All participants were informed of the study's purpose, requirements, and procedures. Each provided written informed consent. The study protocol was approved by the Medical Ethics Committee of Ningbo University (Approval Number: TY2024025) and registered with the Chinese Clinical Trial Registry (Registration Number: ChiCTR2600124657).

### 5.2 Experimental Protocol and Procedures

To ensure that the EMG recordings reflected meaningful neuromuscular activation patterns, a drop‐landing task was selected. This movement imposes high mechanical demands on the lower limb and consistently recruits the muscle groups responsible for ankle stabilization, providing an appropriate basis for synergy extraction. Clinically, drop‐landing is relevant to CAI because impaired neuromuscular control during landing is a common mechanism underlying ankle sprain. All participants were required to perform the task using their dominant leg, defined as the preferred leg for kicking a ball or stepping up. For the CAI cohorts, only individuals whose self‐reported affected ankle was on the dominant side were included. Consequently, the affected leg was consistently the dominant leg for all enrolled CAI patients. Healthy controls similarly performed the test on their dominant leg to maintain rigorous comparability. Before the formal experiment, all subjects were given 15 min to warm up and familiarize themselves with the drop landing maneuver. To avoid influencing their natural landing pattern, no feedback or instruction on landing technique was provided. During the execution phase, subjects stood on a 40‐cm‐high jump box positioned directly in front of the force plate, with their arms crossed in front of their chest. Upon hearing the “start” signal, they moved the leading leg forward, leaned forward, and descended vertically without initial velocity. Subjects were instructed to land on their dominant leg as close to the center of the force plate as possible and maintain balance on one leg (Figure 6a). A trial was considered successful if the subject could maintain balance on the dominant leg for three seconds without signs of falling. Each subject completed 5 successful landing trials. The landing phase was defined as the period from the initial ground contact to the moment of maximum knee flexion.

**FIGURE 6:**
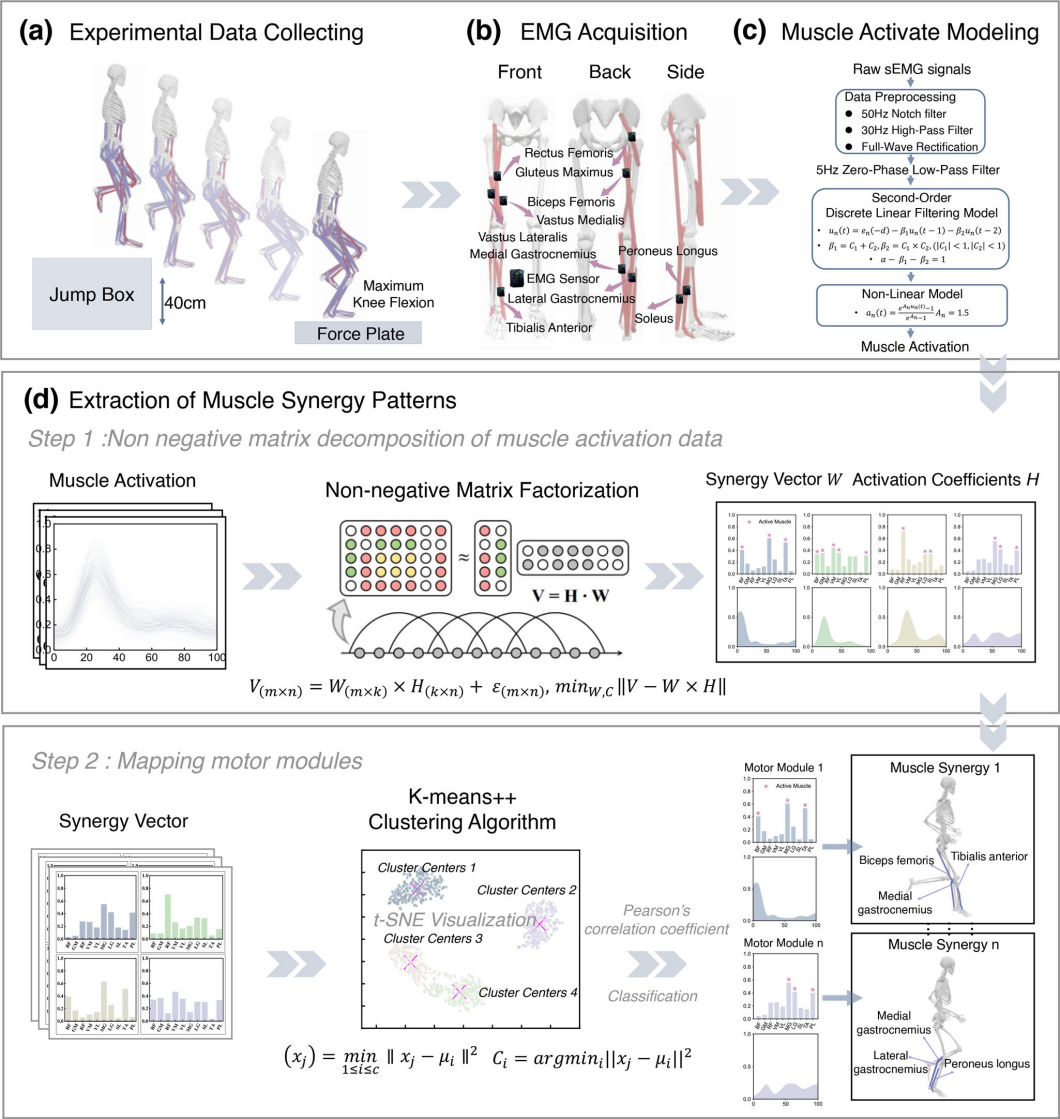
Workflow for muscle synergy analysis. (a) Experimental paradigm. (b) sEMG recordings of 10 lower limb muscles acquired in a controlled laboratory environment. (c) Muscle activations estimated using an auto‐encoded second‐order recursive filtering model, serving as input for NNMF. (d) Synergy patterns extracted by decomposing muscle activations through NNMF, followed by clustering into motor modules using the K‐means++ algorithm.

### 5.3 Data Acquisition and Pre‐Processing

In this study, a 16‐channel Delsys wireless surface EMG system (Delsys, Boston, USA) was used to record sEMG signals from the lower limb muscles during the landing maneuver, with a sampling frequency of 1000 Hz (Figure 6b). Ten muscles of the dominant limb were analyzed, including the BF, GM, RF, VM, VL, MG, LG, SL, TA, and PL. EMG electrodes were placed, and signals acquired following SENIAM guidelines [54]. Optimal electrode sites were identified via isometric or flexion‐extension tasks to localize the largest proportion of the muscle belly. Skin was cleaned with alcohol and shaved if needed to reduce impedance. A single trained experimenter ensured consistent electrode placement, aligning sensors parallel to the muscle fiber direction. Subjects then performed an in situ longitudinal jump to verify signal quality and check for displacement. Electrodes were secured with adhesive tape and a protective skin film to prevent movement artifacts. The subjects then performed maximal voluntary contraction (MVC) trials for each muscle. EMG windows were extracted using a custom MATLAB script. Initial contact was identified from vertical ground reaction forces exceeding 20 N on the force plate (Kistler, Winterthur, Switzerland), while peak knee flexion was determined from knee joint kinematics obtained via a Vicon motion capture system (Vicon Metrics Ltd., UK). All systems were hardware‐synchronized, and EMG signals were resampled and time‐aligned with motion‐capture data prior to segmentation.

To extract physiologically relevant activation signals from raw surface sEMG data, we implemented a custom muscle activation modeling pipeline using MATLAB R2022 (MathWorks, MA, USA) (Figure 6c). Raw sEMG signals were first preprocessed by applying a 50 Hz notch filter to suppress power‐line interference, followed by a 30 Hz zero‐phase high‐pass filter to remove low‐frequency motion artifacts, and full‐wave rectification. The rectified signal was then smoothed using a 5 Hz zero‐phase low‐pass filter to emulate the low‐pass characteristics of muscle activation. The same preprocessing procedure was applied to MVC trials to determine the peak sEMG value, which served as the normalization reference. Processed sEMG signals during the jumps were divided by this MVC peak to yield normalized EMG waveforms. Muscle activation was subsequently computed using a second‐order recursive filtering model, which incorporated a 10 *ms* time delay to account for electromechanical coupling and recursively solved neural excitation from the normalized EMG input [55]. Model stability was constrained using fixed recursive parameters, and a nonlinear transformation was applied to convert neural excitation into muscle activation. A shape coefficient, termed activation nonlinearity coefficient, was set to 1.5 to parameterize the nonlinear excitation‐activation mapping [56]. Finally, all activation waveforms were rescaled to 101 data points corresponding to 0%–100% of the movement cycle. Figure S4 presents the waveform data of muscle activation estimated by the muscle activation model.

### 5.4 Muscle Synergy Pattern Extraction

This study employed NNMF to decompose each subject's processed muscle activation matrix into two non‐negative components: synergy vectors (*W*) capturing spatial coordination patterns, and activation coefficients (*C*) reflecting temporal dynamics [57]. The decomposition was solved as a constrained optimization problem by minimizing the Euclidean reconstruction error under non‐negativity constraints, using a multiplicative update scheme within a gradient‐based iterative framework. The optimal number of synergies (*N_opt_*) was determined using a VAF–based criterion, a widely adopted metric for quantifying reconstruction fidelity. For each candidate number of synergies, both global VAF and muscle‐specific VAFs were computed. Following established protocols, the minimal number of synergies was selected such that the global VAF exceeded 90% while all individual muscle VAFs surpassed 75%, ensuring both overall and localized reconstruction integrity [25, 58].

To extract canonical motor modules, all synergy vectors from healthy participants were concatenated and subjected to unsupervised clustering using the K‐means++ algorithm. This initialization‐optimized variant of K‐means improves clustering consistency by probabilistically selecting initial centroids in proportion to the squared distance from the nearest existing centroid [59, 60]. After initializing centroids, the algorithm iteratively refined cluster assignments and centroid positions until convergence. The resulting cluster centroids were defined as reference motor modules representative of typical neuromuscular coordination patterns in healthy individuals. Subsequently, each synergy vector obtained under various test conditions was matched to its most similar reference module based on Pearson correlation. A correlation threshold (*r* > 0.6) was used to assign vectors to their corresponding module class, and their associated activation coefficients were categorized accordingly, enabling interpretable module‐level tracking across conditions. An overview of the NNMF‐based synergy extraction and module classification pipeline is provided in Figure 6d. All procedures were implemented in MATLAB (MathWorks, Natick, MA, USA) using the built‐in “nnmf” and “kmeans” functions. Hyperparameter settings for both algorithms are detailed in Table S6.

### 5.5 Multi‐Domain Feature Extraction

To enable robust classification of neuromuscular control patterns, we extracted a comprehensive multi‐domain feature set from muscle synergy decomposition.

Spatial features were derived from the synergy vector matrix, which encodes the relative muscle contributions within each synergy module. The *F_w_*, constructed by vectorizing this matrix, captures the geometric distribution of muscle weighting across modules. To further encapsulate functional modularity, a binarized functional structure feature (*F_wf_*) was defined by thresholding muscle weights at 0.5, thereby isolating task‐specific recruitment patterns that reflect physiological sparsity. Inter‐individual consistency in spatial organization was quantified via the OVR, computed as the slope from a linear regression of muscle indices against their corresponding synergy weights [61].

Temporal dynamics were characterized using activation coefficients that reflect time‐dependent synergy recruitment. The average activation coefficient was defined as the mean signal amplitude spanning from the first to the last zero‐crossing within the activation profile. Building upon this baseline, we extracted key temporal features including *T_start_*, *T_end_*, *T_duration_*, *T_peak_*, and mean activation amplitude (*P_Average_*). *T_start_* was defined as the earliest time point at which the activation coefficient exceeded the mean activation amplitude, whereas *T_end_* was defined as the last time point at which the coefficient remained above this threshold. *T_duration_* was calculated as the temporal interval between *T_start_* and *T_end_*. *T_peak_* corresponded to the time at which the maximum activation occurred within the same profile. Complementing these, canonical time‐domain metrics including Mean Absolute Value (MAV), VAR, Integrated Electromyography (IEMG), Root Mean Square (RMS), Zero‐Crossing Rate (ZCR), Waveform Length (WL), and Slope Sign Change (SSC) were calculated to comprehensively capture signal magnitude, variability, and temporal complexity, all indicative of motor effort and control fidelity. Given the inherently nonlinear and complex characteristics of neuromuscular signals, we incorporated entropy‐based metrics such as Sample Entropy (SampEn), Approximate Entropy (ApEn), and Fuzzy Entropy (FuzzyEn) to assess the irregularity and predictability of synergy activations [62, 63]. These metrics were uniformly computed using an embedding dimension of 2 and a similarity threshold set to 20% of the activation signal's standard deviation, ensuring methodological consistency across subjects [64].

Spectral characteristics were assessed via Fourier transform‐based frequency analysis. Activation signals underwent detrending and windowing to minimize artifacts and spectral leakage, followed by zero‐padding to standardize frequency resolution. From the resultant power spectral density, we extracted MDF, Mean Frequency (MNF), Total Power (TP), and PF, collectively describing dominant oscillatory components and energy distribution.

### 5.6 Hierarchical Multi‐Domain Fusion Classification Model

To address the limitations in current motor function assessments for patients with ankle sprains—such as cumbersome procedures, ceiling effects in subjective assessments, time consumption, and limited accuracy—a hierarchical multi‐domain fusion classification framework was developed based on a random forest (RF) classification algorithm, integrating decision information from both temporal and spatial domain muscle activation classifiers (Figure 7). The RF classification model was developed using scikit‐learn (v1.5.1) in Python (v3.12.7) within a standard Anaconda environment.

**FIGURE 7:**
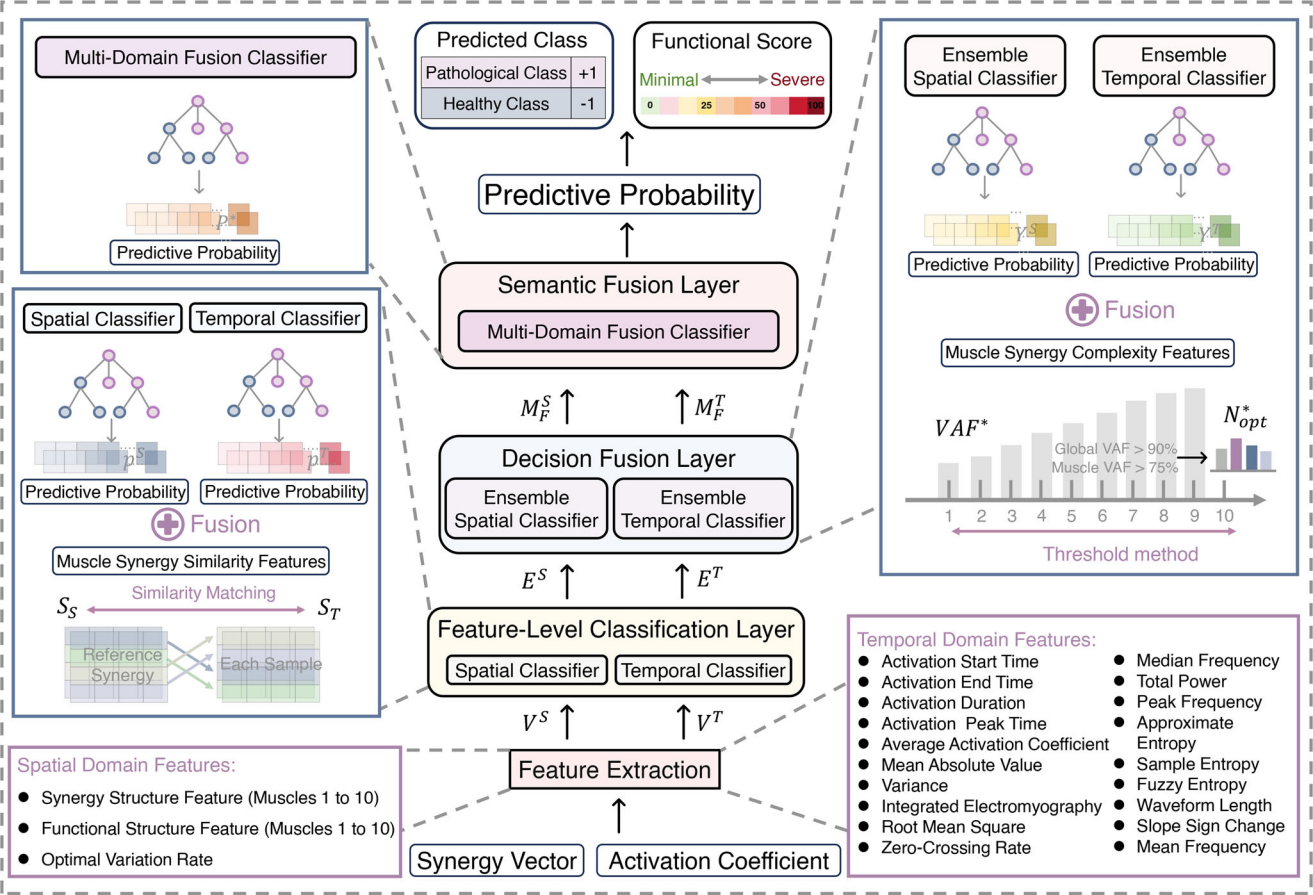
Schematic of the proposed data‐driven hierarchical multi‐domain classification model for individualized and dynamic evaluation of motor function.

We first developed a spatial classifier and a temporal classifier. For the spatial classifier, the input vector *V^S^* was constructed by integrating *F_w_*, *F_wf_*, and ω.

(1) $$V^{S}=\left[F_{w},F_{wf,\;}\;\omega \right]$$

For the temporal classifier, the input vector *V^T^* was constructed by combining 19 time‐domain features, including *T_start_*, *T_end_*, *T_duration_*, *T_peak_*, *P_Average_*, MAV, VAR, IEMG, RMS, ZC, WL, SSC, SampEn, FuzzyEn, ApEn, MDF, MNF, TP, PF, among others.

(2) $$V^{T}=\begin{array}{c} {}[T_{start},T_{end},T_{duration},T_{peak},\;P_{Average},\;MAV,VAR,\\ \mathrm{IEMG},\;\mathrm{RMS},\;\mathrm{ZC},\;\mathrm{WL},\;\mathrm{SSC},\;\mathrm{SampEn},\;\mathrm{FuzzyEn},\;\mathrm{ApEn},\;\mathrm{MDF},\;\mathrm{MNF},\;\mathrm{TP},\;\mathrm{PF}] \end{array}\;$$

The group membership of each participant was used to assign ground truth labels to the input vectors, with samples from the pathological group labeled as +1 and those from the healthy group as ‐1. Subsequently, domain‐specific feature vectors were input into their respective classifiers to generate the corresponding prediction outputs. The probability values *p^S^*, *p^T^* generated by the RF model quantify the position of an individual's motor features along a continuous spectrum ranging from healthy to pathological states.

(3) $$p^{S}=f^{S}\;\left(V^{S}\right)=\frac{1}{N}\;\sum_{i=1}^{N}h_{i}^{S}\left(V^{S}\right)$$

(4) $$p^{T}=f^{T}\;\left(V^{T}\right)=\frac{1}{N}\;\sum_{i=1}^{N}h_{i}^{T}\left(V^{T}\right)$$

where f* denotes the RF classifier, $h_{i}^{*}$ denotes the output of the *i*‐th decision tree, and *N* is the total number of trees in the forest.

Feature fusion and decision fusion are employed as dual‐modal optimization strategies in pattern recognition systems to improve model performance at both the feature representation and decision‐making levels [43]. Compared to employing either feature fusion or decision fusion independently, a hybrid fusion framework that integrates both approaches generally yields superior classification performance [65]. Accordingly, the ensemble spatial/temporal classifiers proposed in this study incorporate the decision‐level outputs from spatial/temporal classifier, along with muscle synergy similarity features, to construct the final input vector. The corresponding integration strategy is formulated as follows:

(5) $$E^{S}=\left[p^{S},S_{S}\right]\;$$

(6) $$E^{T}=\left[p^{T},S_{T}\right]\;$$

where *S_S_* and *S_T_* represent the similarity scores calculated as the Pearson correlation coefficients between the synergy vectors /activation coefficients of interest and the corresponding baseline synergy vectors /activation coefficients. The baseline synergy vectors and activation coefficients are derived from cluster centroids obtained through the K‐means clustering algorithm. *E^S^* and *E^T^* denote the concatenation of decision output vectors and the corresponding similarity scores derived from the spatial/temporal classifiers, respectively. These concatenated vectors serve as the input to ensemble spatial/temporal classifiers. Accordingly, the final output vector of the ensemble classifier can be uniformly formulated as:

(7) $$Y^{S}=f_{ensemble}^{S}\;\left(E^{S}\right)$$

(8) $$Y^{T}=f_{ensemble}^{T}\;\left(E^{T}\right)$$

where Y* denotes the vector of predicted probabilities indicating the likelihood that each trial sample belongs to the pathological group. After independently constructing the ensemble spatial and temporal classifiers, a multi‐domain fusion scheme was employed to establish the final ensemble model. The input vectors of this model incorporate the predicted outputs from both ensemble spatial and temporal classifiers, along with the muscle synergy complexity features, as defined below:

(9) $$M_{F}^{S}=\left[\begin{array}{c} Y^{S},VAF^{S},\;N_{opt}^{S} \end{array}\right]\;$$

(10) $$M_{F}^{T}=\left[\begin{array}{c} Y^{T},VAF^{T},\;N_{opt}^{T} \end{array}\right]\;$$

where $N_{opt}^{*}$ denotes the optimal number of muscle synergies, and VAF* represents the global variance accounted for, which effectively characterizes the redundancy and coordination of muscle control. This measure provides a sound physiological interpretation and offers valuable discriminatory power for classification tasks. Accordingly, the prediction output of the multi‐domain fusion classification model can be formulated as follows:

(11) $$P=G_{fusion}\;\left(M_{F}^{S},M_{F}^{T}\right)$$

where *P* denotes the final predicted probability of a sample being classified as belonging to the pathological group in each trial, and *G_fusion_* represents the multi‐domain fusion framework, which integrates the decision outputs of the ensemble spatial and temporal classifiers along with muscle synergy complexity features.

The output probability value *P* of the model‐estimated likelihood that a given trial exhibits characteristics consistent with CAI, quantified by its similarity to the CAI feature distribution in the learned space. To assess the overall functional status of each subject, the predicted probabilities across all trials were averaged, and the resulting mean was linearly transformed into an integer‐based functional score ranging from 0 to 100. This approach effectively extends the original discrete classification task into a continuous probability domain, thereby enabling a more nuanced characterization of individual motor function degradation. The resulting scores are intuitively interpretable: higher values suggest a functional state more aligned with pathology, whereas lower values indicate a state closer to normal motor function.

### 5.7 Establishment of Clinical Thresholds and Functional Grading

Data‐driven metrics and clinical gold‐standard anchoring were integrated to establish diagnostic and severity cut‐offs for the continuous motor function score. ROC analysis of the model outputs was employed to define the absolute diagnostic boundary, maximizing Youden's Index to differentiate normal from pathological states. To delineate actionable severity intervals, a robust mapping between the algorithmic outputs and the FAAM was established via linear regression. Guided by established clinical benchmarks, thresholds for mild and severe functional impairment were anchored at FAAM scores of 90, based on the reported minimal clinically important difference [14], and 75, according to previously reported clinical criteria [66], respectively. Ninety‐five percent prediction intervals were calculated to quantify the associated mapping uncertainty. The discriminant validity of these thresholds was corroborated by stratifying the cohort according to the severity criteria and evaluating the differences in raw muscle synergy features across the defined groups.

### 5.8 Training Method for Classification Model

The training strategy for the classification model was implemented via a multi‐stage cascaded approach. Initially, a total of 500 valid sEMG trials were obtained from the primary cohort of 50 patients with CAI and 50 healthy controls, after excluding invalid recordings (e.g., due to evident post‐landing instability or poor signal quality). The dataset was partitioned subject‐wise into a training set (60% of the subjects) and an independent test set (40% of the subjects). The training set was further partitioned into three mutually exclusive subsets in a 4:3:3 ratio. Specifically, the first subset (40% of the training data) was used to train the base spatial and temporal classifiers. These trained base classifiers then predicted the class probabilities for the second subset (30%). These probabilities, concatenated with similarity‐based features, were used to train the intermediate ensemble spatial and temporal classifiers. Subsequently, the third subset (30%) was used to train the final multi‐domain fusion classifier by integrating muscle synergy complexity features with the outputs from the intermediate ensemble models.

During each subset training stage, the hyperparameters of the RF classifiers were systematically optimized using a grid search combined with internal fivefold stratified cross‐validation. Based on established guidelines [67, 68], the hyperparameter search space encompassed the number of estimators, maximum tree depth, minimum samples per split, and minimum samples per leaf. The GridSearchCV module in the Scikit‐learn library was employed to identify the optimal parameter configuration based on the highest macro‐averaged F1‐score. Finally, the optimized multi‐domain fusion model was evaluated on the independent held‐out test set. To further evaluate the generalizability of the optimized classifier across CAI subtypes, the trained model was directly evaluated on an expanded subtype‐stratified dataset without additional training. This dataset comprised 100 patients with CAI, including 50 patients with MAI and 50 patients with FAI. Multi‐class classification performance and the reliability of the continuous functional score were systematically evaluated across these subgroups.

### 5.9 Model Evaluation and Interpretability

The performance of the proposed classification models was evaluated using accuracy, precision, recall, F1‐measure, and the AUC‐ROC. Specifically, for the multi‐class evaluation, overall accuracy and macro‐averaged metrics (macro‐precision, macro‐recall, macro F1‐measure, and macro‐AUC), along with confusion matrices, were employed to ensure a balanced assessment across all clinical subtypes. The fidelity of the interpretable surrogate regression model was assessed by quantifying its agreement with the continuous functional scores generated by the proposed machine learning framework. The R^2^, Pearson correlation coefficient (r), RMSE, and MAE were calculated to evaluate the accuracy of surrogate approximation.

Model interpretability was further characterized using SHAP, a post hoc interpretability framework grounded in cooperative game theory that assigns additive importance values to individual features based on their marginal contributions to model predictions [69, 70]. SHAP analysis was applied to the trained classifiers across the hierarchical framework to identify influential neuromuscular features contributing to classification decisions and to visualize how specific feature values affected individual predictions. Furthermore, these analyses provided insights into the contribution of feature‐level and decision‐level fusion strategies within the proposed framework. An interpretable post hoc surrogate framework was established to characterize the relationship between input features and the continuous functional score. Specifically, a tree‐based regression model was trained using the original input features to approximate the continuous functional scores generated by the proposed machine learning framework. SHAP dependence analysis was subsequently applied to this surrogate model to quantify nonlinear feature–score relationships and identify feature‐level threshold effects associated with clinically defined severity boundaries.

### 5.10 Statistical Analysis

All statistical analyses were conducted using SPSS software (version 25.0; IBM Corp., Armonk, NY, USA) and the open‐source SPM1d package. Continuous variables were presented as mean ± standard deviation. The assumptions of data normality and homogeneity of variance were evaluated using the Shapiro–Wilk test and Levene's test, respectively. To investigate the statistical differences in the extracted discrete features between the ankle instability cohorts and healthy controls, independent‐samples t‐tests were conducted. In instances where the data violated the assumptions of normality or equal variance, the non‐parametric Wilcoxon rank‐sum test was applied. All p‐values were adjusted for multiple comparisons using the Bonferroni correction. Furthermore, for the continuous temporal activation coefficients spanning the entire drop‐landing cycle, one‐dimensional Statistical Parametric Mapping (SPM1d) was utilized to rigorously quantify and locate specific movement phases with significant inter‐group divergence. The threshold for statistical significance was defined a priori at *p* < 0.05.

## Author Contributions

**Tianle Jie**: conceptualization, methodology, investigation, validation, visualization, writing – original draft. **Datao Xu**: conceptualization, methodology, investigation, visualization, validation, writing – original draft. **Zhifeng Zhou**: methodology, visualization, validation, writing – review and editing. **Bas Van Hooren**: validation, writing – original draft, writing – review and editing. **Huiyu Zhou**: methodology, visualization, validation, writing – review and editing. **Yi Yuan**: methodology, supervision, writing – review and editing. **Xiangli Gao**: investigation, visualization. **Monèm Jemni**: methodology, validation, writing – review and editing. **Liangliang Xiang**: investigation, validation, writing – review and editing. **Meizi Wang**: investigation, validation, writing – review and editing. **Justin Fernandez**: methodology, validation. **Tibor J. Goda**: methodology, validation, supervision. **Yaodong Gu**: conceptualization, supervision, funding acquisition, writing – review and editing.

## Funding

This research was supported by the National Key Research and Development Program of China (2024YFC3607305), Zhejiang Provincial Natural Science Foundation of China for Distinguished Young Scholars (LR22A020002), Zhejiang Provincial Key Research and Development Program of China (2023C03197), Ningbo key R&D Program (2022Z196), Zhejiang Engineering Research Center for New Technologies and Applications of Helium‐Free Magnetic Resonance Imaging Open Fund Project 2024 (2024GCLX06), National Natural Science Foundation of China (12502378), and China Postdoctoral Science Foundation (2026M793490).

## Conflicts of Interest

The authors declare no conflicts of interest.

## Clinical Trial Registration

The study protocol was retrospectively registered with the Chinese Clinical Trial Registry (Registration Number: ChiCTR2600124657).

## Supporting information




**Supporting File**: advs77212‐sup‐0001‐SuppMat.docx.

## Data Availability

The complete dataset supporting the findings of this study has been deposited in Figshare and is publicly available at https://doi.org/10.6084/m9.figshare.29595887.v1. Source data underlying the figures and analyses are provided within the article and its Supporting Information. The code used for model development, training, and evaluation is openly available at https://github.com/TianleJie/CAI‐FuncScoreNet.
