# Supplementary material for: An Interpretable, Data‐Driven, Hierarchical Multi‐Domain Fusion Framework for Classification and Motor Function Scoring in Chronic Ankle Instability
Source: Adv Sci (Weinh). 2026 Aug 31;13(55):e77212. doi: 10.1002/advs.77212 (PMC13528686; doi:10.1002/advs.77212)
Supplement: Supplementary file 1 [file ADVS-13-e77212-s001.docx]

Supporting Information

An interpretable, data-driven, hierarchical multi-domain fusion framework for classification and motor function scoring in chronic ankle instability

Tianle Jie, Datao Xu, Zhifeng Zhou, Bas Van Hooren, Huiyu Zhou, Yi Yuan, Xiangli Gao, Monèm Jemni, Liangliang Xiang, Meizi Wang, Justin Fernandez, Tibor J. Goda, Yaodong Gu^*^

**This Supporting information file includes:**

Table S1: Statistical comparison muscle VAFs between CAI and healthy groups across synergy orders (1–9)

Table S2: t-SNE algorithm hyperparameter configuration

Table S3: Statistical comparison of extracted muscle synergy features between CAI and healthy groups

Table S4: Statistical comparison of muscle synergy complexity features between CAI and Healthy Groups

Table S5: Comparison with related research

Table S6: Parameter settings for NNMF and K-means++ algorithms

Figure S1: Spatial synergy structures and temporal activation dynamics of the four extracted neuromuscular control modules

Figure S2: Comprehensive visualization of multi-domain feature sets used in machine learning models

Figure S3: Hyperparameter importance evaluation and three-dimensional performance landscapes across classification models

Figure S4: Continuous waveform visualization of muscle activation profiles across different ankle instability subtypes and healthy controls

Supporting Information Text S1: Detailed analysis of spatial synergy structures and temporal activation dynamics

References

**Table S1.** Statistical comparison muscle VAFs between CAI and healthy groups across synergy orders (1–9).

|  | CAI  Mean (SD) | Healthy  Mean (SD) | p-value |
| --- | --- | --- | --- |
| **Muscle VAFs with one synergy (%)** |  |  |  |
| biceps femoris | 45.90(6.95) | 62.66(7.53) | <0.001 |
| gluteus maximus | 65.80(9.17) | 65.16(7.82) | 0.398 |
| rectus femoris | 49.17(6.08) | 61.59(6.48) | <0.001 |
| vastus medialis | 85.65(4.59) | 77.90(5.86) | <0.001 |
| vastus lateralis | 67.34(8.18) | 76.80(4.99) | <0.001 |
| medial gastrocnemius | 71.51(5.78) | 68.65(5.30) | <0.001 |
| lateral gastrocnemius | 76.71(4.44) | 87.47(3.77) | <0.001 |
| soleus | 72.10(7.29) | 74.02(5.86) | 0.001 |
| tibialis anterior | 50.31(7.99) | 44.25(6.83) | <0.001 |
| peroneus longus | 77.29(6.03) | 74.97(5.66) | <0.001 |
| **Muscle VAFs with two synergies (%)** |  |  |  |
| biceps femoris | 77.70(7.97) | 75.82(5.58) | 0.002 |
| gluteus maximus | 70.79(8.23) | 68.08(7.75) | <0.001 |
| rectus femoris | 82.14(6.91) | 78.14(5.69) | <0.001 |
| vastus medialis | 86.52(4.60) | 83.12(5.46) | <0.001 |
| vastus lateralis | 72.22(7.85) | 80.42(4.27) | <0.001 |
| medial gastrocnemius | 82.64(5.93) | 84.11(4.22) | <0.001 |
| lateral gastrocnemius | 78.02(3.42) | 89.58(3.22) | <0.001 |
| soleus | 76.21(7.94) | 86.54(5.14) | <0.001 |
| tibialis anterior | 76.43(8.69) | 77.75(7.77) | 0.076 |
| peroneus longus | 79.12(5.31) | 78.55(5.65) | 0.241 |
| **Muscle VAFs with three synergies (%)** |  |  |  |
| biceps femoris | 84.76(4.94) | 84.50(4.72) | 0.544 |
| gluteus maximus | 85.03(4.78) | 84.74(5.84) | 0.548 |
| rectus femoris | 95.84(1.77) | 90.80(3.70) | <0.001 |
| vastus medialis | 91.10(3.81) | 91.94(2.50) | 0.004 |
| vastus lateralis | 84.54(5.70) | 88.12(4.12) | <0.001 |
| medial gastrocnemius | 89.61(2.85) | 92.61(2.29) | <0.001 |
| lateral gastrocnemius | 80.20(3.23) | 91.98(2.86) | <0.001 |
| soleus | 88.67(3.88) | 90.28(3.36) | <0.001 |
| tibialis anterior | 87.11(3.96) | 85.69(4.55) | <0.001 |
| peroneus longus | 89.91(3.62) | 81.75(4.37) | <0.001 |
| **Muscle VAFs with four synergies (%)** |  |  |  |
| biceps femoris | 91.09(3.40) | 93.93(2.21) | <0.001 |
| gluteus maximus | 88.63(3.31) | 89.17(3.36) | 0.072 |
| rectus femoris | 97.07(1.37) | 96.02(1.67) | <0.001 |
| vastus medialis | 93.49(1.95) | 93.23(2.30) | 0.185 |
| vastus lateralis | 90.42(3.02) | 89.12(4.20) | <0.001 |
| medial gastrocnemius | 91.53(2.62) | 95.83(1.68) | <0.001 |
| lateral gastrocnemius | 92.46(3.32) | 94.51(1.91) | <0.001 |
| soleus | 92.82(2.64) | 92.71(2.58) | 0.639 |
| tibialis anterior | 90.94(3.23) | 90.68(3.79) | 0.401 |
| peroneus longus | 91.70(3.03) | 91.80(3.19) | 0.712 |
| **Muscle VAFs with five synergies (%)** |  |  |  |
| biceps femoris | 93.60(2.60) | 94.77(1.90) | <0.001 |
| gluteus maximus | 91.96(3.28) | 92.00(3.15) | 0.884 |
| rectus femoris | 97.91(1.24) | 97.07(1.40) | <0.001 |
| vastus medialis | 94.98(1.74) | 95.33(1.79) | 0.026 |
| vastus lateralis | 92.69(2.70) | 93.27(2.80) | 0.019 |
| medial gastrocnemius | 95.48(2.09) | 97.03(1.29) | <0.001 |
| lateral gastrocnemius | 94.73(2.24) | 95.47(1.71) | <0.001 |
| soleus | 94.06(2.02) | 93.79(2.38) | 0.169 |
| tibialis anterior | 93.47(2.71) | 93.80(2.49) | 0.157 |
| peroneus longus | 93.57(2.33) | 94.81(2.44) | <0.001 |
| **Muscle VAFs with six synergies (%)** |  |  |  |
| biceps femoris | 95.33(1.82) | 95.46(1.95) | 0.408 |
| gluteus maximus | 94.68(2.71) | 93.99(2.61) | 0.004 |
| rectus femoris | 97.98(1.25) | 97.65(1.23) | 0.003 |
| vastus medialis | 96.20(1.31) | 96.47(1.46) | 0.027 |
| vastus lateralis | 94.80(2.09) | 95.46(2.10) | <0.001 |
| medial gastrocnemius | 96.92(1.61) | 97.80(0.87) | <0.001 |
| lateral gastrocnemius | 96.12(1.76) | 96.64(1.36) | <0.001 |
| soleus | 95.44(1.59) | 95.47(1.94) | 0.835 |
| tibialis anterior | 95.33(2.10) | 95.35(2.02) | 0.899 |
| peroneus longus | 95.11(2.07) | 96.04(1.85) | <0.001 |
| **Muscle VAFs with seven synergies (%)** |  |  |  |
| biceps femoris | 96.27(1.89) | 96.38(1.78) | 0.481 |
| gluteus maximus | 96.76(1.79) | 95.98(2.00) | <0.001 |
| rectus femoris | 97.73(1.52) | 97.94(1.22) | 0.094 |
| vastus medialis | 97.14(1.16) | 97.22(1.31) | 0.488 |
| vastus lateralis | 95.83(2.05) | 96.50(1.87) | <0.001 |
| medial gastrocnemius | 97.78(1.15) | 98.13(0.90) | <0.001 |
| lateral gastrocnemius | 97.30(1.35) | 97.75(0.98) | <0.001 |
| soleus | 96.34(1.47) | 96.60(1.58) | 0.063 |
| tibialis anterior | 96.65(1.77) | 96.23(1.91) | 0.011 |
| peroneus longus | 96.49(1.67) | 96.92(1.74) | 0.006 |
| **Muscle VAFs with eight synergies (%)** |  |  |  |
| biceps femoris | 96.86(1.84) | 97.25(1.44) | 0.010 |
| gluteus maximus | 97.55(1.62) | 97.11(1.71) | 0.003 |
| rectus femoris | 97.87(1.45) | 98.04(1.18) | 0.168 |
| vastus medialis | 97.97(1.09) | 97.79(1.15) | 0.067 |
| vastus lateralis | 96.84(1.87) | 97.54(1.23) | <0.001 |
| medial gastrocnemius | 98.25(0.92) | 98.55(0.64) | <0.001 |
| lateral gastrocnemius | 98.02(1.05) | 98.33(0.74) | <0.001 |
| soleus | 97.17(1.26) | 97.23(1.49) | 0.625 |
| tibialis anterior | 97.27(1.50) | 97.08(1.75) | 0.196 |
| peroneus longus | 97.76(1.24) | 97.70(1.20) | 0.610 |
| **Muscle VAFs with nine synergies (%)** |  |  |  |
| biceps femoris | 97.56(1.80) | 98.01(1.30) | 0.001 |
| gluteus maximus | 98.16(1.30) | 97.93(1.44) | 0.060 |
| rectus femoris | 98.02(1.33) | 98.25(1.10) | 0.037 |
| vastus medialis | 98.63(0.71) | 98.35(0.93) | <0.001 |
| vastus lateralis | 97.77(1.57) | 98.20(1.15) | <0.001 |
| medial gastrocnemius | 98.66(0.76) | 98.88(0.60) | <0.001 |
| lateral gastrocnemius | 98.47(0.93) | 98.77(0.57) | <0.001 |
| soleus | 98.01(1.10) | 97.97(1.20) | 0.674 |
| tibialis anterior | 97.89(1.41) | 98.00(1.37) | 0.373 |
| peroneus longus | 98.38(0.93) | 98.18(1.37) | 0.06 |

**Table S2.** t-SNE algorithm hyperparameter configuration.

| Parameter | Value | Description |
| --- | --- | --- |
| Output Dimensions | 2 | Dimensionality of the low-dimensional embedding space |
| Distance Metric | Euclidean | Similarity computed using Euclidean distance in high-dimensional space |
| Perplexity | 30 | Effective number of local neighbors considered in similarity calculation |
| Learning Rate | 200 | Controls the step size during gradient descent optimization |
| Number of Iterations | 1000 | Total number of optimization iterations |
| Initialization Method | PCA-based | Initial coordinates in low-dimensional space determined by PCA |

**Table S3.** Statistical comparison of extracted muscle synergy features between CAI and healthy groups.

| Features | Muscle Synergy 1 | | | Muscle Synergy 2 | | | Muscle Synergy 3 | | | Muscle Synergy 4 | | |
| --- | --- | --- | --- | --- | --- | --- | --- | --- | --- | --- | --- | --- |
|  | CAI | Healthy | p-value | CAI | Healthy | p-value | CAI | Healthy | p-value | CAI | Healthy | p-value |
| $F_{w}^{BF}$ | 0.05±0.05 | 0.03±0.04 | 0.013 | 0.03±0.04 | 0.11±0.12 | <0.001 | 0.52±0.13 | 0.50±0.13 | 0.021 | 0.16±0.09 | 0.47±0.13 | <0.001 |
| $F_{w}^{\mathrm{GM}}$ | 0.16±0.10 | 0.05±0.07 | <0.001 | 0.06±0.07 | 0.11±0.12 | <0.001 | 0.12±0.08 | 0.21±0.09 | <0.001 | 0.42±0.12 | 0.51±0.11 | <0.001 |
| $F_{w}^{\mathrm{RF}}$ | 0.18±0.21 | 0.35±0.24 | <0.001 | 0.98±0.06 | 0.95±0.08 | <0.001 | 0.02±0.03 | 0.07±0.05 | <0.001 | 0.07±0.07 | 0.17±0.14 | <0.001 |
| $F_{w}^{\mathrm{VM}}$ | 0.30±0.12 | 0.32±0.17 | 0.321 | 0.33±0.11 | 0.37±0.15 | 0.003 | 0.20±0.09 | 0.13±0.10 | <0.001 | 0.46±0.11 | 0.66±0.15 | <0.001 |
| $F_{w}^{\mathrm{VL}}$ | 0.08±0.09 | 0.22±0.10 | <0.001 | 0.21±0.13 | 0.23±0.12 | 0.075 | 0.06±0.04 | 0.18±0.12 | <0.001 | 0.34±0.12 | 0.50±0.11 | <0.001 |
| $F_{w}^{\mathrm{MG}}$ | 0.52±0.15 | 0.66±0.17 | <0.001 | 0.29±0.13 | 0.28±0.18 | 0.462 | 0.54±0.16 | 0.80±0.15 | <0.001 | 0.17±0.12 | 0.20±0.14 | 0.05 |
| $F_{w}^{\mathrm{LG}}$ | 0.40±0.14 | 0.51±0.18 | <0.001 | 0.30±0.13 | 0.47±0.20 | <0.001 | 0.17±0.09 | 0.32±0.11 | <0.001 | 0.26±0.20 | 0.44±0.14 | <0.001 |
| $F_{w}^{\mathrm{SL}}$ | 0.10±0.09 | 0.22±0.14 | <0.001 | 0.24±0.12 | 0.45±0.15 | <0.001 | 0.07±0.05 | 0.06±0.05 | <0.001 | 0.39±0.12 | 0.43±0.14 | 0.003 |
| $F_{w}^{\mathrm{TA}}$ | 0.13±0.10 | 0.17±0.10 | 0.076 | 0.10±0.08 | 0.07±0.07 | <0.001 | 0.54±0.13 | 0.65±0.13 | <0.001 | 0.06±0.07 | 0.04±0.05 | <0.001 |
| $F_{w}^{\mathrm{PL}}$ | 0.22±0.10 | 0.49±0.13 | <0.001 | 0.21±0.12 | 0.22±0.15 | 0.432 | 0.13±0.06 | 0.07±0.07 | <0.001 | 0.42±0.13 | 0.48±0.16 | <0.001 |
| $F_{wf}^{\mathrm{BF}}$ | 0.00±0.00 | 0.00±0.00 | / | 0.00±0.00 | 0.01±0.08 | 0.157 | 0.54±0.49 | 0.51±0.50 | 0.478 | 0.00±0.00 | 0.43±0.49 | <0.001 |
| $F_{wf}^{\mathrm{GM}}$ | 0.00±0.00 | 0.00±0.00 | / | 0.00±0.00 | 0.03±0.17 | 0.004 | 0.00±0.00 | 0.01±0.06 | 0.318 | 0.24±0.43 | 0.52±0.50 | <0.001 |
| $F_{wf}^{\mathrm{RF}}$ | 0.11±0.32 | 0.27±0.44 | 0.356 | 1.00±0.00 | 1.00±0.00 | / | 0.00±0.00 | 0.00±0.00 | / | 0.01±0.09 | 0.03±0.18 | 0.049 |
| $F_{wf}^{\mathrm{VM}}$ | 0.02±0.17 | 0.15±0.36 | 0.368 | 0.05±0.23 | 0.21±0.40 | <0.001 | 0.00±0.00 | 0.01±0.06 | 0.318 | 0.34±0.47 | 0.85±0.35 | <0.001 |
| $F_{wf}^{\mathrm{VL}}$ | 0.00±0.00 | 0.01±0.07 | 0.080 | 0.02±0.16 | 0.03±0.18 | 0.563 | 0.00±0.00 | 0.02±0.14 | 0.025 | 0.06±0.24 | 0.49±0.50 | <0.001 |
| $F_{wf}^{\mathrm{MG}}$ | 0.55±0.50 | 0.81±0.38 | 0.635 | 0.06±0.25 | 0.17±0.37 | <0.001 | 0.58±0.49 | 0.97±0.15 | <0.001 | 0.02±0.15 | 0.04±0.21 | 0.208 |
| $F_{wf}^{\mathrm{LG}}$ | 0.20±0.41 | 0.50±0.50 | 0.619 | 0.08±0.27 | 0.42±0.49 | <0.001 | 0.01±0.06 | 0.07±0.26 | <0.001 | 0.20±0.40 | 0.34±0.47 | 0.003 |
| $F_{wf}^{\mathrm{SL}}$ | 0.00±0.00 | 0.05±0.22 | 0.262 | 0.04±0.21 | 0.39±0.48 | <0.001 | 0.00±0.00 | 0.00±0.00 | / | 0.17±0.37 | 0.27±0.44 | 0.025 |
| $F_{wf}^{\mathrm{TA}}$ | 0.00±0.00 | 0.00±0.00 | / | 0.00±0.00 | 0.00±0.00 | / | 0.62±0.48 | 0.89±0.30 | <0.001 | 0.00±0.00 | 0.00±0.00 | / |
| $F_{wf}^{\mathrm{PL}}$ | 0.00±0.00 | 0.43±0.49 | 0.944 | 0.03±0.17 | 0.04±0.21 | 0.323 | 0.00±0.00 | 0.00±0.00 | / | 0.25±0.43 | 0.45±0.49 | <0.001 |
| OVR | 0.01±0.01 | 0.03±0.01 | <0.001 | -0.01±0.01 | -0.01±0.01 | 0.079 | 0.0018±0.0075 | 0.0025±0.010 | 0.003 | 0.0040±0.0095 | -0.017±0.015 | <0.001 |
| $T_{start}$ | 9.54±7.74 | 20.74±10.90 | <0.001 | 29.97±4.36 | 21.66±3.85 | <0.001 | 1.08±0.68 | 1.00±0.00 | 0.050 | 12.37±2.47 | 9.98±2.06 | <0.001 |
| $T_{end}$ | 83.65±8.13 | 98.19±8.10 | <0.001 | 88.32±13.00 | 70.40±22.19 | <0.001 | 46.73±34.21 | 49.18±36.25 | 0.436 | 46.80±20.39 | 40.30±12.60 | 0.001 |
| $T_{duration}$ | 44.88±7.32 | 52.80±7.11 | <0.001 | 46.86±7.30 | 34.52±7.64 | <0.001 | 24.70±5.74 | 22.15±4.19 | <0.001 | 25.70±3.14 | 26.13±4.00 | 0.263 |
| $T_{peak}$ | 28.65±21.73 | 55.82±31.34 | <0.001 | 44.12±5.89 | 34.20±5.94 | <0.001 | 8.23±1.97 | 6.96±1.77 | <0.001 | 23.32±3.14 | 21.44±1.14 | <0.001 |
| $P_{Average}$ | 0.44±0.10 | 0.57±0.08 | <0.001 | 0.47±0.05 | 0.38±0.08 | <0.001 | 0.27±0.03 | 0.26±0.04 | 0.318 | 0.25±0.02 | 0.25±0.03 | 0.306 |
| MAV | 0.42±0.09 | 0.56±0.08 | <0.001 | 0.47±0.05 | 0.38±0.08 | <0.001 | 0.27±0.03 | 0.26±0.04 | 0.314 | 0.25±0.02 | 0.24±0.02 | <0.001 |
| VAR | 0.06±0.01 | 0.06±0.01 | 0.525 | 0.08±0.01 | 0.07±0.01 | <0.001 | 0.08±0.01 | 0.08±0.01 | <0.001 | 0.08±0.01 | 0.08±0.01 | 0.118 |
| IEMG | 43.23±9.71 | 57.35±9.04 | <0.001 | 47.52±5.89 | 38.44±8.98 | <0.001 | 27.51±3.49 | 27.16±4.28 | 0.314 | 25.68±2.75 | 24.54±2.77 | <0.001 |
| RMS | 0.50±0.08 | 0.62±0.08 | <0.001 | 0.55±0.04 | 0.47±0.06 | <0.001 | 0.39±0.02 | 0.39±0.02 | 0.005 | 0.38±0.02 | 0.37±0.02 | 0.062 |
| ZC | 1.17±0.96 | 0.77±0.90 | 0.019 | 1.09±1.02 | 0.91±1.05 | 0.046 | 1.05±1.29 | 0.75±1.29 | 0.009 | 1.46±1.27 | 1.93±1.25 | <0.001 |
| WL | 3.05±0.49 | 2.80±0.59 | 0.013 | 2.24±0.29 | 2.22±0.23 | 0.600 | 1.62±0.28 | 1.54±0.18 | <0.001 | 2.34±0.15 | 2.23±0.20 | <0.001 |
| SSC | 7.67±2.01 | 7.74±2.31 | 0.870 | 5.98±1.42 | 6.34±1.96 | 0.018 | 8.15±2.21 | 8.91±2.17 | <0.001 | 7.29±1.77 | 7.50±2.05 | 0.329 |
| SampEn | 0.0047±0.0030 | 0.0052±0.0025 | 0.387 | 0.0037±0.0024 | 0.0047±0.0028 | <0.001 | 0.0086±0.0011 | 0.0083±0.0010 | 0.002 | 0.0050±0.0027 | 0.0044±0.0027 | 0.067 |
| FuzzyEn | 2.75±0.16 | 2.75±0.21 | 0.987 | 2.59±0.09 | 2.49±0.16 | <0.001 | 1.97±0.22 | 1.85±0.19 | <0.001 | 2.20±0.13 | 2.21±0.14 | 0.508 |
| ApEn | 0.0043±0.0037 | 0.0050±0.0038 | 0.319 | 0.0048±0.0026 | 0.0049±0.0030 | 0.569 | 0.0045±0.0022 | 0.0050±0.0027 | 0.041 | 0.0057±0.0027 | 0.0041±0.0029 | <0.001 |
| MDF | 4.10±1.80 | 6.79±1.19 | <0.001 | 3.98±1.01 | 3.26±1.34 | <0.001 | 7.45±0.99 | 8.03±0.86 | <0.001 | 3.31±0.94 | 2.65±0.77 | <0.001 |
| MNF | 4.63±1.86 | 7.39±0.87 | <0.001 | 5.29±1.06 | 4.17±1.64 | <0.001 | 7.78±0.68 | 8.16±0.51 | <0.001 | 4.10±1.13 | 3.14±1.09 | <0.001 |
| PF | 4.58±0.86 | 5.80±0.82 | <0.001 | 4.53±0.48 | 3.99±0.51 | <0.001 | 5.31±0.47 | 5.50±0.42 | <0.001 | 3.93±0.20 | 3.78±0.18 | <0.001 |
| TP | 0.21±0.02 | 0.25±0.01 | <0.001 | 0.20±0.02 | 0.19±0.02 | <0.001 | 0.27±0.01 | 0.28±0.01 | <0.001 | 0.20±0.02 | 0.18±0.01 | <0.001 |

**Table S4.** Statistical comparison of muscle synergy complexity features between CAI and healthy groups.

| Features | Group | |  |
| --- | --- | --- | --- |
|  | CAI | Healthy | p-value |
| $N_{\mathrm{opt}}$ | 3.96±0.31 | 3.78±0.46 | <0.05 |
| $S_{S}$ | 0.39±0.26 | 0.45±0.29 | <0.001 |
| $S_{T}$ | 0.45±0.27 | 0.45±0.33 | 0.006 |
| ${VAF}_{1}$ | 64.5±2.63 | 69.6±2.01 | <0.001 |
| ${VAF}_{2}$ | 79.2±2.58 | 81.1±1.76 | <0.001 |
| ${VAF}_{3}$ | 88.0±1.50 | 88.8±1.61 | <0.001 |
| ${VAF}_{4}$ | 92.3±1.21 | 93.1±1.31 | <0.001 |
| ${VAF}_{5}$ | 94.5±0.95 | 95.0±0.98 | <0.001 |
| ${VAF}_{6}$ | 96.0±0.80 | 96.3±0.78 | <0.001 |
| ${VAF}_{7}$ | 96.9±0.76 | 97.1±0.75 | 0.001 |
| ${VAF}_{8}$ | 97.6±0.80 | 97.7±0.68 | 0.008 |
| ${VAF}_{9}$ | 98.1±0.71 | 98.2±0.66 | 0.011 |

Note: $N_{\mathrm{opt}}$ denotes the optimal number of extracted muscle synergies. $S_{S}$ and $S_{T}$ represent the spatial and temporal similarity scores, respectively, calculated as Pearson correlation coefficients relative to the healthy baseline. $\mathrm{VA}F_{1}$ to $\mathrm{VA}F_{9}$ indicate the Variance Accounted For corresponding to synergy orders ranging from 1 to 9 during the non-negative matrix factorization (NNMF) process.

**Table S5.** Comparison with related research.

| Ref. | Task | Input Features | Classifier | Prediction Target | Performance |
| --- | --- | --- | --- | --- | --- |
| Hwang et al. [1] (2024) | Heel rise test | Age, work duration, body mass index, calcaneal stance position angle in the initial and terminal positions, calcaneal movement, plantar flexion angle | Random Forest | Binary classification: CAI vs. Non-CAI | AUC = 0.967 |
|  |  |  |  |  | Accuracy = 92.5% |
|  |  |  |  |  | F1-score = 88.9% |
|  |  |  |  |  | Recall = 1.000 |
| Gu et al. [2] (2024) | Treadmill running gait analysis | 18-channel 3D joint kinematics: XYZ coordinates of 6 joints (right/left hip, knee, ankle) | GaitNet + ARL (Graph Neural Network + Attention Reinforcement Learning) | Binary classification: CAI vs. Control | Accuracy: 96% |
|  |  |  |  |  | AUC: 1.00 |
| Hwang et al. [3] (2025) | Daily work of couriers | ankle strength, range of motion, postural control, anatomical deformities | Support Vector Machine | Binary classification (CAI vs. non-CAI) | AUC = 0.817 |
|  |  |  |  |  | Accuracy = 75% |
|  |  |  |  |  | F1 = 74.9% |
|  |  |  |  |  | Precision = 75% |
| Kamachi et al. [4] (2025) | Ultrasound imaging of the anterior talofibular ligament under anterior drawer stress | Ultrasound images of ATFL under maximum anterior stress, cropped to include lateral malleolus, ATFL, and talus | ResNet-50 | Binary classification (CLAI vs. control) | AUC = 0.998 |
|  |  |  |  |  | Accuracy = 0.989 |
|  |  |  |  |  | Precision = 1.00 |
|  |  |  |  |  | Recall = 0.980 |
|  |  |  |  |  | F-Measure = 0.990 |
| Hwang et al. [5] (2025) | Assessing postural control, ankle ROM, muscle strength, and anatomical deformities in parcel delivery workers | BMI, work duration, YBT composite score, ankle dorsiflexion ROM, lunge angle, evertor/invertor strength ratios, navicular drop, Resting Calcaneal Stance Position, Eyes Closed Single Leg Stance success/retrials | Random Forest | Binary classification (CAI vs. non-CAI) | AUC = 0.853 |
|  |  |  |  |  | F1 = 0.747 |
|  |  |  |  |  | Precision = 0.839 |
|  |  |  |  |  | Recall = 0.792 |
| Liu et al. [6] (2022) | Gait analysis (walking) | Spatiotemporal (gait phase percentages, stride length/duration) and kinematic (ankle ROM, velocity variables) | Convolutional LSTM | Binary classification (CAI vs. control) | AUC = 1.00 |
|  |  |  |  |  | Accuracy = 100% |
|  |  |  |  |  | F1 = 1.00 |
| Qian et al. [7] (2020) | Running gait analysis | Clustering coefficients from sparse subspace clustering of 18 joint motion channels (hip, knee, ankle) | Linear SVM | Binary classification (CAI vs. control) | >70% accuracy (hit rate and majority voting accuracy) |
| Yin et al. [8] (2025) | Ankle MRI analysis | MRI scans (lateral/medial collateral ligament images) | Transformer-based AnkleNet (multilabel Deep Learning) | CAI diagnosis (including RCAI) | Macro-average AUC = 0.870 |
|  |  |  |  |  | Balanced accuracy = 0.805 |
| Guo et al. [9] (2025) | Level walking gait analysis | Multi-modal sensor data:8 FSR plantar pressure sensors, 3-axis accelerometer + gyroscope, Center of Pressure trajectory, Time-to-Boundary metrics, | XGBoost | Binary classification: CAI vs. Control | Accuracy: 93.39% |
|  |  |  |  |  | AUC: 0.959 |

**Table S6.** Parameter settings for NNMF and K-means++ algorithms.

| Algorithm | Parameter Name | Value | Description |
| --- | --- | --- | --- |
| **NNMF** | MaxIter | 200 | Maximum number of iterations for convergence. |
|  | TolFun | 1e-4 | Tolerance on the objective function for convergence. |
|  | Replicates | 20 | Number of independent runs with random initializations. |
|  | Algorithm | 'mult' | Multiplicative update algorithm for non-negative matrix factorization. |
| **K-means ++** | Distance | 'cosine' | Cosine distance metric for clustering. |
|  | Replicates | 15 | Number of clustering attempts with different centroid seeds. |

**Figure S1.** Spatial synergy structures and temporal activation dynamics of the four extracted neuromuscular control modules. The top row illustrates the muscle synergy weights (spatial domain) for each module across the CAI, FAI, MAI, and Healthy groups. Asterisks denote statistically significant differences between specific groups based on post-hoc analysis (* $p<0.05$, ** $p<0.01$, *** $p<0.001$). The bottom row displays the continuous activation coefficients over the entire drop-landing movement cycle (0–100%). The horizontal bars at the bottom of each temporal plot highlight specific phases of the movement cycle where SPM1d identified significant main effects among the cohorts. Abbreviations: BF, biceps femoris; GM, gluteus maximus; LG, lateral gastrocnemius; MG, medial gastrocnemius; PL, peroneus longus; RF, rectus femoris; SL, soleus; TA, tibialis anterior; VL, vastus lateralis; VM, vastus medialis.

**Figure S2.** Comprehensive visualization of the multi-domain feature sets utilized in the classification models. For the functional structure features ($F_{wf}$), each horizontal track corresponds to a specific muscle within a synergy vector. Vertical blue bars denote active muscles (value = 1), whereas blank spaces indicate inactive muscles (value = 0). The features $VAF_{1}$ to $VAF_{9}$ represent the Variance Accounted For corresponding to synergy orders ranging from 1 to 9 during the NNMF process. The variables $P^{S}$, $Y^{S}$, $P^{T}$, $Y^{T}$, and $P^{fusion}$ denote the predicted probabilities of classifying a sample as pathological, derived from the base spatial, ensemble spatial, base temporal, ensemble temporal, and multi-domain fusion classifiers, respectively. Abbreviations: ApEn, Approximate Entropy; FuzzyEn, Fuzzy Entropy; IEMG, Integrated EMG; MAV, Mean Absolute Value; MDF, Median Frequency; MNF, Mean Frequency; OVR, Optimal Variation Rate; PF, Peak Frequency; RMS, Root Mean Square; SampEn, Sample Entropy; SSC, Slope Sign Change; $S_{S}$, Spatial similarity score; $S_{T}$, Temporal similarity score; TP, Total Power; VAR, Variance; WL, Waveform Length; ZC, Zero Crossing.

**Figure S3.** Hyperparameter importance evaluation and three-dimensional performance landscapes across classification models. (a) Relative importance of key hyperparameters identified through grid search. (b) Three-dimensional performance landscapes illustrating the interaction effects among maximum tree depth (x-axis), minimum samples per leaf (y-axis), and number of estimators (z-axis) on overall classification accuracy, with the color gradient representing the accuracy level. From left to right, the columns correspond to the base spatial, ensemble spatial, base temporal, ensemble temporal, and multi-domain fusion classifiers, respectively. Abbreviations: MD, maximum tree depth; MSL, minimum samples per leaf; MSS, minimum samples split; NE, number of estimators.

**Figure S4**. Continuous waveform visualization of muscle activation profiles across different ankle instability subtypes and healthy controls. The panels display the activation amplitudes over the normalized landing phase (0–100%) for the (a) Chronic Ankle Instability, (b) Functional Ankle Instability, (c) Mechanical Ankle Instability, and (d) Healthy control groups. Within each group, the activation patterns of the ten evaluated lower-limb muscles are presented.

**Supporting Information Text S1**: Detailed Analysis of Spatial Synergy Structures and Temporal Activation Dynamics

Further detailed comparisons of the extracted synergy structures and activation coefficients across subgroups revealed distinct motor strategies (**Figure S1**). In Synergy Module 1, the CAI, FAI, and MAI groups exhibited significantly higher weights in BF and lower weights in MG compared to the Healthy group (p < 0.001). Additionally, TA weights were significantly elevated in the CAI and MAI groups (p < 0.01), while no significant difference was observed between the FAI and Healthy groups. Synergy Module 2 involved the VM, VL, and GM muscles. The Healthy group demonstrated significantly greater weights in both VM and VL than the three ankle instability groups (p < 0.001). For the GM muscle, only the FAI group showed a significant decrease ·compared to the Healthy controls (p < 0.05), with no statistical differences found for the CAI and MAI groups. In Synergy Module 3, RF weights remained consistently high across all cohorts with no significant group differences. Conversely, the weights for LG and SL demonstrated a substantial and significant reduction across the CAI, FAI, and MAI cohorts (p < 0.001), decreasing from approximately 0.5 in the Healthy group to below 0.3. Finally, Synergy Module 4 revealed notable variations among the PL, LG, and MG muscles. The weights of the PL muscle were significantly reduced in the CAI, FAI, and MAI groups compared to the higher levels maintained by the Healthy group (p < 0.001). Furthermore, MG weights were significantly greater in all three instability groups than in the Healthy group (FAI: p < 0.001; CAI: p < 0.01; MAI: p < 0.05). Regarding the LG muscle, only the MAI group presented a significantly higher weight than the Healthy group (p < 0.001), with the CAI and FAI groups showing no significant differences.

In the temporal domain, SPM1d was employed to precisely quantify continuous differences in the activation coefficients across the entire landing cycle. In Synergy Module 1, the CAI, FAI, and MAI groups showed fluctuating activation levels compared to the Healthy group. Specifically, all three instability groups exhibited significantly lower activation immediately after initial contact (0%-10%) and during the mid-portion of the landing phase (40%–51%). However, they demonstrated significantly higher activation during the early (11%–32%), mid-to-late (53%–73%), and terminal phases (82%-100%) (p < 0.001). For Synergy Module 2, the three instability groups demonstrated significantly reduced activation during the early (4%–28%) and mid-to-late (50%–68%) stages of the drop landing. Conversely, these groups maintained significantly higher activation levels during the intermediate phase (28%–50%) and the final phase (69%-100%) (p < 0.001). Synergy Module 3 exhibited a distinct divergence in activation. The CAI, FAI, and MAI groups showed significantly decreased activation during the early landing phase (5%–38%). After this period, their activation levels became significantly higher than the Healthy group and remained elevated throughout the rest of the landing phase (40%–99%) (p < 0.001). In Synergy Module 4, the three instability cohorts displayed significantly higher activation during the early phase following initial contact (3%–30%). Subsequently, their activation was significantly reduced compared to the Healthy baseline throughout the middle and late phases of the landing movement (35%–99%) (p < 0.001).

**References**

1.	U. J. Hwang, O. Y. Kwon, J. H. Kim, G. T. Gwak, "Classification of chronic ankle instability using machine learning technique based on ankle kinematics during heel rise in delivery workers," *Digit Health* 10(2024): 20552076241235116, https://doi.org/10.1177/20552076241235116.

2.	H. Gu, S. C. Yen, E. Folmar, C. A. Chou, "GaitNet+ARL: A Deep Learning Algorithm for Interpretable Gait Analysis of Chronic Ankle Instability," *IEEE J Biomed Health Inform* 28, no. 7 (2024): 3918, https://doi.org/10.1109/JBHI.2024.3383588.

3.	U. J. Hwang, O. Y. Kwon, J. H. Kim, G. T. Gwak, "Machine learning for classifying chronic ankle instability based on ankle strength, range of motion, postural control and anatomical deformities in delivery service workers with a history of lateral ankle sprains," *Musculoskelet Sci Pract* 75(2025): 103230, https://doi.org/10.1016/j.msksp.2024.103230.

4.	M. Kamachi, K. Kamada, N. Kanzaki, et al., "Using deep learning for ultrasound images to diagnose chronic lateral ankle instability with high accuracy," *Asia Pac J Sports Med Arthrosc Rehabil Technol* 40(2025): 1, https://doi.org/10.1016/j.asmart.2025.01.001.

5.	U. J. Hwang, O. Y. Kwon, J. H. Kim, G. T. Gwak, "Factors contributing to chronic ankle instability in parcel delivery workers based on machine learning techniques," *BMC Med Inform Decis Mak* 25, no. 1 (2025): 80, https://doi.org/10.1186/s12911-025-02919-7

6.	X. Liu, C. Zhao, B. Zheng, et al., "Spatiotemporal and kinematic characteristics augmentation using Dual-GAN for ankle instability detection," *Math Biosci Eng* 19, no. 10 (2022): 10037, https://doi.org/10.3934/mbe.2022469.

7.	S. Qian, S.-C. Yen, E. Folmar, C.-A. Chou, "Self-expressive subspace clustering to recognize motion dynamics for chronic ankle instability," *IISE Transactions on Healthcare Systems Engineering* 10, no. 1 (2020): 60, https://doi.org/10.1080/24725579.2019.1673521

8.	R. Yin, H. Chen, C. Wang, et al., "Transformer-based multilabel deep learning model is efficient for detecting ankle lateral and medial ligament injuries on magnetic resonance imaging and improving clinicians’ diagnostic accuracy for rotational chronic ankle instability," *Arthroscopy: The Journal of Arthroscopic & Related Surgery* 41, no. 3 (2025): 574, https://doi.org/10.1016/j.arthro.2024.05.027

9.	Z. Guo, Y. Li, Y. Wang, et al., "Intelligent Diagnosis and Predictive Rehabilitation Assessment of Chronic Ankle Instability Using Shoe-Integrated Sensor System," *IEEE Trans Neural Syst Rehabil Eng* 33(2025): 1978, https://doi.org/10.1109/TNSRE.2025.3563924.
